# Actin cortex formation is required for integrin clustering but not activation during leukocyte adhesion

**DOI:** 10.7150/ijbs.137102

**Published:** 2026-09-10

**Authors:** Doulathunnisa Ahamed Younis, Raphael Serna, Wei Liu, Beiyan Zhou, Anthony T. Vella, Liang Hu, Yunfeng Chen, Ji Yu, Emanuela M. Bruscia, Zhichao Fan

**Affiliations:** 1 Department of Immunology, School of Medicine, UConn Health, 263 Farmington Ave., Farmington, Connecticut 06030, USA.; 2 Academy of Integrative Medicine, Shanghai University of Traditional Chinese Medicine, 1200 Cai Lun Road, Shanghai 201203, China.; 3 Department of Biochemistry and Molecular Biology and Department of Pathology, University of Texas Medical Branch, 301 University Blvd, Galveston, Texas 77555, USA.; 4 Center for Cell Analysis and Modeling, UConn Health, 263 Farmington Ave., Farmington, Connecticut 06030, USA.; 5 Department of Pediatrics, Yale School of Medicine, New Haven, Connecticut 06510, USA.

**Keywords:** actin cortex, monocyte adhesion, β_2_ integrins, integrin clustering, integrin activation, CORO1A

## Abstract

Leukocyte recruitment is critical for tissue homeostasis and inflammatory responses. Chemokine-induced β_2_ integrin-dependent adhesion is the first and most critical step of leukocyte recruitment. Previous studies have shown that crosstalk between integrins and the actin cytoskeleton is vital for the migration of adherent leukocytes. However, whether and how the actin cytoskeleton contributes to integrin-dependent leukocyte adhesion remains poorly defined. As we recently demonstrated, a defect in integrin clustering, even in the presence of normal integrin activation, is sufficient to cause an adhesion defect in cystic fibrosis monocytes. Interestingly, these defects were accompanied by a concomitant deficiency in actin cortex formation, suggesting that actin cortex formation specifically facilitates integrin clustering rather than activation. In this study, we test this hypothesis by using the pharmacological actin polymerization inhibitor cytochalasin D (CytoD) in both human and mouse monocytes. Interestingly, we found that CytoD selectively impaired chemokine-induced actin cortex formation, yet had no impact on the total amount of polymerized F-actin. Using super-resolution microscopy, we found that inhibiting actin cortex formation directly suppressed integrin clustering but not integrin activation. Cells with inhibited actin cortex formation prevented Coronin 1A (CORO1A) binding to integrins, suggesting that the actin cortex is critical for CORO1A recruitment to integrins and thereby facilitates integrin clustering. Expanding beyond the integrin-activation-centric leukocyte adhesion paradigm, we underscore the importance of integrin clustering and identify that the actin cortex specifically regulates integrin clustering but not activation. This fundamental finding warrants investigation into selective targeting in the design of therapeutic strategies for inflammation.

## Introduction

The recruitment of leukocytes, including monocytes and neutrophils, from the bloodstream is crucial in controlling infections and regulating inflammation 1-4. Leukocyte recruitment occurs in a cascade-like fashion 3, 5, 6, including the initiation of rolling on vascular endothelium, chemokine-induced firm adhesion, spreading on the endothelial surface, intravascular crawling, transmigration through vascular endothelium, and in-tissue migration. β_2_ integrins are heterodimeric adhesion molecules expressed on leukocytes. The β_2_ integrin-dependent conversion from rolling to firm adhesion is a critical step in this cascade 3, 7-11. During this conversion, G protein-coupled receptors (GPCRs, e.g., CXCR1/2 on neutrophils 12 or CCR2 on monocytes 13) expressed on leukocytes are stimulated by chemokines (e.g., CXCL1/2 and IL-8 for neutrophils 14 or CCL2 for monocytes 13), triggering a signaling pathway known as integrin inside-out signaling 15, 16. This integrin inside-out signaling induces conformational changes in the β_2_ integrin ectodomain, increasing its adhesiveness to ligands, a process called integrin “activation” 5, 11, 14, 17-19.

After firm adhesion, integrin-ligand interactions trigger another signaling pathway, integrin outside-in signaling 16, amplifying integrin activation and inducing changes in leukocyte morphology that facilitate spreading on the endothelial surface and enable intravascular crawling. Previous studies using culture models with adherent cells also showed that integrin outside-in signaling can induce the “clustering” of integrin molecules 20-23, in which cooperative interactions support larger forces than those of sporadic integrins. Our recent study showed that, before integrin outside-in signaling is involved, integrin inside-out signaling also increases integrin clustering 13, 24. More importantly, we showed that integrin clustering induced by inside-out signaling is essential for leukocyte adhesion and is regulated independently of integrin activation. For example, we showed that monocytes deficient in cystic fibrosis transmembrane conductance regulator (CFTR) exhibited an integrin clustering defect but not an integrin activation defect, resulting in an adhesion defect 13. In another study, we showed that leukocytes lacking talin-1 exhibit an integrin activation defect but not an integrin clustering defect 24. These two studies suggested that integrin activation and clustering induced by inside-out signaling are regulated through different mechanisms. Given the novelty of the updated model of integrin-mediated cell adhesion, the underlying mechanisms regulating these two processes, especially clustering, remain largely unclear.

The actin cytoskeleton is critical for leukocyte recruitment in multiple steps. During rolling, actin polymerization is an essential component of tethers 25 and slings 26, stabilizing the adhesion molecule anchors. After leukocyte adhesion, integrin-ligand binding initiates outside-in signaling to induce actin polymerization 27, which contributes to the formation of protrusive cell structures, such as pseudopods, at the front of migrating leukocytes 28. The actin cytoskeleton exerts force, which is also critical for integrin outside-in signaling, dictating integrin orientation during leukocyte migration 29. Defective β-actin was associated with migration deficiencies in both human 30 and mouse 31 leukocytes. Migrating leukocytes also form actin-rich structures called podosomes, which are essential to support cell adhesion 32, matrix degradation 32, 33, and transcellular diapedesis 34. However, how the actin cytoskeleton contributes to the integrin inside-out signaling-dependent transition from leukocyte rolling to firm adhesion is rarely studied. As mentioned above, our previous study determined that integrin clustering is essential for the transition from leukocyte rolling to firm adhesion 13, 24. We also found that integrin clustering-deficient monocytes exhibit a defect in forming the actin cortex 13, a layer of polymerized F-actin approximately adjacent to the cell membrane, suggesting that the actin cortex is critical for inside-out signaling-induced integrin clustering and thereby contributes to leukocyte firm adhesion.

In this study, we found that the pharmacological actin polymerization inhibitor cytochalasin D (CytoD) selectively disrupts chemokine-induced actin cortex formation in both human and mouse monocytes without measurably reducing total F-actin levels. Using super-resolution stochastic optical reconstruction microscopy (STORM), we quantitatively assessed integrin clustering and provided the first evidence that actin cortex formation is essential for the integrin clustering required for leukocyte adhesion. Further analysis showed that actin cortex-dependent Coronin 1A (CORO1A) membrane recruitment and integrin interactions might be critical for integrin clustering and leukocyte adhesion.

## Results

### CytoD inhibits actin cortex formation but does not change overall F-actin amount in monocytes

Chemokine stimulation induces actin polymerization beneath the cell membrane of leukocytes 13, 24, 35, a process called actin cortex formation. First, we wanted to establish a method to inhibit this chemokine-induced actin cortex formation. CytoD is a cell-permeable mycotoxin that binds to the barbed end of actin filaments, thus inhibiting both the association and dissociation of actin subunits 36 to disrupt both actin polymerization and depolymerization. To assess if CytoD can inhibit monocyte actin cortex formation, we performed epifluorescent transverse images showing the cellular F-actin distribution by phalloidin staining in both mouse (**Fig. 1A**) and human (**Fig. 1B**) monocytes. As expected, CCL2 stimulation induced actin cortex formation in monocytes (**Fig. 1A-B**). To quantify actin cortex formation, we performed radial profile analysis of the images (**Fig. 1C-D**), finding that resting monocytes had F-actin located in the cell center (within 2 μm from the center), and CCL2 stimulation enriches F-actin to the cell membrane to form a cortex (a peak of ~3.3 μm from the center in mouse monocytes and ~4 μm from the center in human monocytes). We also quantified cell membrane-proximal fluorescence intensity (**Fig. 1E-F**) and found a ~22% to ~38% increase in the percentage of F-actin (phalloidin) localized to the cell membrane after CCL2 stimulation. After CytoD treatment, we did not notice a decrease in overall phalloidin staining in monocyte epifluorescent images, but we did observe a defect in actin cortex formation (**Fig. 1A-F**). Consistently, our flow cytometry assessment of phalloidin-stained cells showed that CCL2 did not increase actin polymerization, and CytoD did not reduce total F-actin in mouse (**Fig. 1G**) and human (**Fig. 1H**) monocytes. To rule out a nonspecific effect, we also tested monocyte viability and found no significant effect after CytoD treatment (**Fig. S1**). These results suggested that CCL2 induces dynamic changes in the actin cytoskeleton, depolymerizes existing cytoplasmic F-actin, and polymerizes new F-actin proximal to the cell membrane, thus relocating F-actin to the cell membrane and forming an actin cortex in monocytes. Since CytoD inhibits both actin polymerization and depolymerization, the existing F-actin is immobilized, resulting in a consistent amount of cellular F-actin that fails to relocate to the cell membrane.

### The actin cortex is dispensable for β_2_ integrin activation on monocytes

Since the current model for β_2_ integrin-dependent leukocyte adhesion primarily focuses on integrin activation, we first assessed whether actin cortex formation affects β_2_ integrin activation by testing the binding of conformation-specific reporter antibodies to monocytes using both flow cytometry (**Fig. 2A-D**) and super-resolution STORM microscopy (**Fig. 2E-F**). We used two conformation-specific monoclonal antibodies to report β_2_ integrin activation: mAb24, which reports high ligand-binding affinity (**Fig. 2A, C, E**), and KIM127, which reports ectodomain extension, enabling integrin to bind the ligand in trans (**Fig. 2B, D, F**). Since these antibodies only work for human β_2_ integrins, we used monocytes isolated from human β_2_ integrin knock-in mice 37 (**Fig. 2A-B**) and, importantly, from human blood samples (**Fig. 2C-F**). We found that CCL2 stimulation increased the binding of both mAb24 and KIM127 to monocytes. However, CytoD treatment, which eliminated actin cortex formation (**Fig. 1**), had no effect on mAb24 and KIM127 binding, neither quantified by median fluorescence intensity of monocytes in flow cytometry (**Fig. 2A-D**), nor quantified by molecular localization number per cell in STORM (**Fig. 2E-F**), suggesting that actin cortex formation is not required for β_2_ integrin activation.

### The actin cortex is essential for β_2_ integrin clustering on monocytes

Because our previous studies showed that, in addition to activation, β_2_ integrin clustering is also essential for leukocyte firm adhesion 13, we then assessed whether the actin cortex is required for β_2_ integrin clustering on monocytes using super-resolution STORM microscopy (**Fig. 3**), providing nanoscale single-molecule resolution 38, 39 to quantify molecular clustering 13, 24, 40-42. To ensure monocytes in our assay underwent only CCL2-initiated integrin inside-out signaling, not ligand-binding-initiated outside-in signaling, we kept them in suspension during stimulation and staining to prevent nonspecific surface adhesion, then fixed and immobilized them for imaging. These procedures were distinct from previous clustering studies using adherent-cell culture models 20-23. Both mouse (**Fig. 3A-D**) and human monocytes (**Fig. 3E-K**) were used in our assay for comprehensiveness. After analyzing STORM images of β_2_ integrins on the mouse monocyte surface (the contact area with the coverslip, **Fig. 3A**) using the Voronoi tessellation algorithm 41, 43, we found that CCL2 stimulation significantly increased cluster number (**Fig. 3B**) and β_2_ integrin localizations per cluster (**Fig. 3D**), whereas CytoD treatment eliminated these increases. There was no significant change in cluster density (**Fig. 3C**), suggesting that clusters were larger after CCL2 stimulation and that CytoD treatment eliminated this effect. Consistent results were shown in the cluster analysis using density-based spatial clustering of applications with noise (DBSCAN, **Fig. S2A-C**).

In human monocyte integrin clustering assays, we imaged activated β_2_ integrins using conformational-specific reporter antibodies mAb24 and KIM127 (**Fig. 3E**) to enable more accurate assessment of integrin ligand-binding avidity than staining with pan-β_2_ integrin antibodies. Monocytes from all groups were stimulated with CCL2 to ensure adequate staining with mAb24 and KIM127. Comparable numbers of mAb24 and KIM127 localizations were found between CytoD-treated and control monocytes (**Fig. 2E-F**), indicating the integrin availability or labeling was not affected. Consistent with those observed in mouse monocytes (**Fig. 3A-D**), we found that CytoD treatment significantly reduced cluster number per cell (**Fig. 3F, I**) and integrin localization number per cluster (**Fig. 3H, K**) without affecting cluster density (**Fig. 3G, J**) in human monocytes after cluster analysis using both the Voronoi tessellation algorithm and DBSCAN (**Fig. S2D-I**). Taken together, our results demonstrate that the actin cortex is essential for leukocytes to assemble more and larger β_2_ integrin clusters, rather than altering integrin expression or affinity, to support adhesion.

### The actin cortex recruits CORO1A to the cell membrane in monocytes

Since CORO1A is an actin-binding protein that interacts with β_2_ integrins 13, 44 and contributes to neutrophil adhesion, actin cortex formation might be required for CORO1A membrane recruitment, making CORO1A spatially proximal to and able to bind to β_2_ integrins. To test these, we first assessed CCL2-induced CORO1A membrane recruitment in both mouse (**Fig. 4A**) and human (**Fig. 4B**) monocytes using high-resolution epifluorescent imaging. Radial profile analysis (**Fig. 4C-D**) showed that resting monocytes had CORO1A randomly distributed in the cytoplasm (within ~3 μm from the center), and CCL2 stimulation induced the recruitment of CORO1A to the cell membrane (~3 μm from the center in mouse monocytes and ~3.7 μm from the center in human monocytes). Cell membrane proximal fluorescence intensity quantification (**Fig. 4E-F**) showed that the percentage of CORO1A localized around the cell membrane significantly increased from ~20% to ~35% after CCL2 stimulation. After CytoD treatment, we observed a defect in CORO1A recruitment to the cell membrane (**Fig. 4A-F**). Thus, our results showed that the actin cortex is essential for the recruitment of CORO1A to the monocyte cell membrane following CCL2 stimulation.

### The actin cortex is critical for the engagement of CORO1A and β_2_ integrins in monocytes

To test whether the actin cortex is involved in the engagement of CORO1A and β_2_ integrins (CD18), we quantified the co-localization of CORO1A with CD18 in monocytes using dual-color super-resolution STORM (**Fig. 5A-F**), providing ~20 nm spatial resolution 45 to assess molecular spatial engagement. We observed more CORO1A/CD18 co-localization after CCL2 stimulation (white in **Fig. 5A** and** D**, the first vs. third columns) in both mouse (**Fig. 5A**) and human (**Fig. 5D**) monocytes. After quantifying STORM images using coordinate-based co-localization (CBC) analysis, we found significant increases in either CD18 co-localized with CORO1A (**Fig. 5B**, from ~30 to ~40% in mouse monocytes; **Fig. 5E**, from ~25 to ~35% in human monocytes) or CORO1A co-localized with CD18 (**Fig. 5C**, from ~35 to ~43% in mouse monocytes; **Fig. 5F**, from ~30 to ~42% in human monocytes) after CCL2 stimulation. However, CytoD treatment significantly inhibited CCL2-induced co-localization of CORO1A/CD18 (**Fig. 5A** and **D**, the second vs. fourth columns, quantifications in **Fig. 5B-C**,** E-F**). We also performed high-resolution dual-color epifluorescence imaging of CORO1A and CD18 in mouse (**Fig. S3A**) and human (**Fig. S3B**) monocytes and quantified the coefficient of CORO1A/CD18 using Pearson's correlation analysis. Consistent with STORM imaging quantification, CCL2 stimulation increased the CORO1A/CD18 coefficient from ~0.7 to ~0.8 in mouse (**Fig. S3C**) and from ~0.4 to ~0.7 in human (**Fig. S3D**) monocytes, which was eliminated in CytoD-treated actin-cortex-deficient monocytes (**Fig. S3C-D**). We also quantified biochemical binding of CD18 and CORO1A using co-immunoprecipitation (**Fig. 5G-H**), showing that CCL2 stimulation doubled CORO1A binding to CD18 in vehicle-treated monocytes, but not in CytoD-treated, actin-cortex-deficient human monocytes. Expression of CD18 and CORO1A in cell lysates before immunoprecipitation was shown in **Fig. S3E**. Overall, our results indicated that the actin cortex is critical for the CCL2-induced engagement of CORO1A with CD18 in monocytes.

For study comprehensiveness, we also tested F-actin (phalloidin staining)/CD18 co-localization using STORM and high-resolution epifluorescence imaging. We found that F-actin/CD18 co-localization is increased after CCL2 stimulation in both mouse (**Fig. 6A-C**, **S4A**, **S4C**) and human (**Fig. 6D-F**, **S4B**, **S4D**) monocytes. CytoD treatment diminished this CCL2-induced co-localization of F-actin and CD18 (**Fig. 6, S4**).

Lastly, we assessed the co-localization of CORO1A and F-actin using STORM and high-resolution epifluorescence imaging. We found that, regardless of CCL2 stimulation or CytoD treatment, CORO1A maintained its association with F-actin in both mouse (**Fig. S5A-C**) and human (**Fig. S5D-F, S6**) monocytes. There were more co-localized CORO1A/F-actin recruited to the cell membrane in control but not actin-cortex-deficient monocytes following CCL2 stimulation. Altogether, these results suggest that CORO1A binds F-actin consistently in monocytes. CCL2 stimulation induces F-actin dynamic changes and relocates F-actin to form the actin cortex, recruiting F-actin-bound CORO1A to the cell membrane. Thus, CORO1A becomes spatially proximal to β_2_ integrins, allowing CORO1A to bind to β_2_ integrins and facilitating integrin clustering. When actin cortex formation is impaired, CORO1A fails to recruit to the cell membrane and bind to β_2_ integrins, thereby inhibiting the ensuing β_2_ integrin clustering.

## Discussion

The actin cytoskeleton is critical for multiple cellular functions, including cell shape and mechanics, cell movement and migration, cell division, intracellular transport, cell adhesion, interaction, and communication 46, 47. Most previous actin studies focus on adherent cells, even when studying immune cells, ignoring the first and critical step of the immune response—the adhesion of immune cells to the vascular endothelium. In this step, most leukocytes, including neutrophils 5, 14, 48, monocytes 13, 49, 50, and effector T cells 51, first roll along the endothelium using PSGL-1 (P-selectin glycoprotein ligand-1) to bind endothelial P- or E-selectin. After chemokine stimulation, leukocytes firmly adhered to the endothelium via β_2_ integrin binding to intercellular adhesion molecule 1 (ICAM-1) on endothelial cells. During this step, how the actin cytoskeleton contributes to the function of the spherical immune cells is rarely studied. Previous work using latrunculin B (Lat B) to inhibit actin polymerization in neutrophils 52 showed unaffected P-selectin-mediated rolling but decreased ICAM-1-mediated adhesion. To further test how actin polymerization affects ICAM-1-mediated adhesion, they used two conformation-specific reporter antibodies, KIM127 and MEM148, to test β_2_ integrin activation. In the canonical switch-blade model of integrin activation 17, the bent resting integrin first extends its ectodomain, followed by the hybrid domain swing-out and ligand binding pocket opening to acquire high affinity (**Fig. S7**). KIM127 and MEM148 report ectodomain extension and hybrid domain swing-out, respectively.

Their results showed that actin polymerization is required for the hybrid domain swing-out but not for ectodomain extension 52. Unfortunately, imaging techniques were not used to assess how Lat B affects actin polymerization or F-actin structures. In comparison, we found that CytoD treatment does not affect the overall amount of F-actin and specifically inhibits chemokine-induced actin cortex formation in monocytes. We also used conformation-specific reporter antibodies, KIM127 and mAb24, which report ectodomain extension and high ligand-binding affinity, and showed that actin cortex formation is dispensable for the CCL2-induced increase in KIM127 and mAb24. Meanwhile, actin cortex formation is required for β_2_ integrin clustering, an essential factor for leukocyte adhesion 13. When comparing our study with the previous study 52, besides the differences in leukocyte cell type and pharmacological drugs, we conclude some interesting points: 1) both studies agree that actin polymerization/cortex is not required for integrin ectodomain extension; 2) actin polymerization/cortex may be required for integrin hybrid domain swing out, but not high ligand-binding affinity, challenging the consequences of integrin conformational changes proposed in the canonical switch-blade mode 17; 3) The adhesion defect observed in the previous study 52 may be attributed to a defect in integrin clustering but not activation. Meanwhile, although we have shown a selective effect of CytoD on actin cortex formation, it remains undetermined whether this pharmacologic inhibition has actin cortex-independent effects on integrin functions and leukocyte adhesion. Further investigation using mice lacking actin polymerization regulators, such as the actin-related protein 2/3 (Arp2/3) complex 49, 50 and the Wiskott-Aldrich syndrome protein (WASP) 53, may provide further insight into how the actin cytoskeleton regulates integrin-mediated leukocyte adhesion during inflammation.

Our results suggested a central role of the actin cortex in integrin clustering. Actually, the actin cortex has been reported to be essential for the clustering of several immune molecules. For example, T cell receptors (TCRs) are clustered on microvilli in both peripheral blood human T cells and differentiated effector T cells 54. After actin polymerization is inhibited with latrunculin A, T cells lose their microvilli on the cell surface, and TCR molecules are no longer clustered 54. Since our previous study also showed enrichment of integrin clusters on neutrophil microvilli 14, and a prior study showed that CytoD and Lat B treatment can reduce microvilli on the monocyte surface 25, these observations raise the possibility that the defect in integrin clustering we observed here may also be due to the loss of microvilli. Further studies are needed to conceptually distinguish between two intertwined mechanisms: whether the actin cortex directly supports integrin clustering via serving as cytoskeletal anchors, or indirectly facilitates it by regulating membrane topology, such as by forming microvilli, thereby geometrically concentrating integrins. Another study using photoactivated localization microscopy (PALM) showed that disrupting actin polymerization with CytoD reduces the clustering of β2-adrenergic receptors in a cell line model 55. Besides the role of the actin cortex in clustering of surface molecules, actin polymerization in the tethers in response to dragging forces stabilizes integrin anchors that mediate monocyte adhesion 25. Whether dragging-force-induced actin polymerization induces greater integrin clustering at the anchors remains to be determined.

The current model for integrin-mediated leukocyte adhesion primarily focuses on integrin activation. Our current study, along with our previous study 13, underlines the importance of integrin clustering in chemokine-induced leukocyte adhesion. More importantly, combining the results of our previous studies 13, 24 and this study, we found that CFTR and the actin cortex are required for integrin clustering but not activation, and talin-1 is required for integrin activation but not clustering, suggesting a model that integrin activation and clustering are distinct processes, each subject to separate regulation, in chemokine-induced integrin-inside-out signaling. These findings challenged the current paradigm that integrin activation and clustering are interlinked 56, 57. Please also note that most previous studies focusing on integrin clustering used adherent cells 20-23, which cannot eliminate the disturbance caused by outside-in signaling induced by integrin-ligand binding.

Our study also suggests that CORO1A is involved in actin-cortex-dependent β_2_ integrin clustering, consistent with a previous study showing that CORO1A-knockout mouse neutrophils exhibit defects in integrin-dependent adhesion and soluble ligand binding 44. Future studies using super-resolution imaging to assess integrin clustering on CORO1A knockout leukocytes will further define the unique role of CORO1A in integrin clustering. Our co-IP experiment shows increased biochemical interaction between CORO1A and β_2_ integrins in human monocytes after chemokine stimulation, consistent with a previous study showing that the purified cytoplasmic tail of β_2_ integrins pulls down CORO1A from neutrophil cell lysates 44. Our previous study 13 and the current study indicate that the interaction between CORO1A and β_2_ integrins depends on CFTR and on actin cortex formation. It remains to be further determined whether the interaction of CORO1A and β_2_ integrins is a direct molecular interaction or part of a larger actin-associated complex. Because CORO1A does not contain traditional integrin-binding motifs such as phosphotyrosine-binding domains 58, 59 or 4.1 band, ezrin, radixin, moesin domains 59, this interaction may be part of a larger association complex.

## Conclusion

Overall, this study provides direct evidence underlining the importance of actin cortex formation in regulating integrin clustering but not conformational activation in monocytes. Together with previous studies 13, 24, we establish a new paradigm in the cell adhesion field: β2 integrin clustering and activation are two distinct processes governed by distinct molecular mechanisms. Further mechanistic studies focusing on integrin clustering may identify molecular targets for selective therapeutics that suppress arterial inflammation while preserving life-saving immune surveillance.

## Materials and Methods

### Reagents

We obtained Roswell Park Memorial Institute medium 1640 (RPMI-1640) without phenol red, phosphate-buffered saline (PBS) without Ca^2+^ and Mg^2+^, and goat serum from Gibco. We obtained human serum albumin (HSA) and fetal bovine serum (FBS) from Gemini Bio Products. We obtained Ficoll-Paque Plus from Cytiva. We obtained paraformaldehyde (PFA), Pacific Blue Annexin V/SYTOX AADvanced apoptosis kit, and Pierce streptavidin magnetic beads from Thermo Fisher Scientific. We obtained Glutaraldehyde (8%) from Electron Microscopy Sciences. We obtained CytoD, dimethyl sulfoxide (DMSO), and poly-L-lysine from Sigma-Aldrich. We obtained Ficoll-Paque Plus from Cytiva. The KIM127 antibody recognizing the β₂ integrin ectodomain extension was purified from hybridoma supernatant (ATCC) by Leinco Technologies. We labeled KIM127 with DyLight 550 or DyLight 650 by using DyLight antibody labeling kits (Thermo Fisher Scientific), according to the manufacturer's instructions. We obtained the allophycocyanin (APC)- and Alexa Fluor (AF) 647-conjugated conformation-specific antibody mAb24 reporting the headpiece-opening of β_2_ integrins, the APC-conjugated anti-mouse CD115 antibody, the AF647-conjugated anti-mouse CD18 antibody, recombinant human and mouse CCL2, red blood cell (RBC) lysis buffer, and the intracellular staining perm wash buffer from BioLegend. We obtained AF568- and AF647-conjugated goat anti-rabbit IgG secondary antibodies and AF568-conjugated phalloidin from Invitrogen. We obtained the AF647-conjugated anti-human CD18 antibody (TS1/18) from LSBio. We obtained unconjugated rabbit anti-human/mouse CORO1A (D6K5B) antibody and unconjugated rabbit anti-human CD18 (D4N5Z) antibody from Cell Signaling. We obtained the biotinylated anti-human CD18 monoclonal antibody from Leinco Technologies. We obtained the EasySep human monocyte enrichment kit without CD16 depletion and the EasySep mouse monocyte isolation kit from STEMCELL Technologies. We obtained the protease inhibitor cocktail Set III EDTA-free from Millipore.

### Ethics approval and consent to participate

All animal experiments were conducted in accordance with the protocol #AP-201438-0128 approved by the UConn Health Institutional Animal Care and Use Committee (IACUC) and complied with the NIH Guide for the Care and Use of Laboratory Animals.

Heparinized peripheral blood samples were obtained from donors without diagnosed diseases after informed consent, under protocol #20-084-2, approved by the Institutional Review Board of UConn Health, and in accordance with the Declaration of Helsinki.

### Mice

We used wild-type C57BL/6J mice from the Jackson Laboratory (#000664) and human β2 integrin knock-in mice from Dr. Klaus Ley (the Immunology Center of Georgia at Augusta University; currently available from the Jackson Laboratory, #037426). We fed mice a standard rodent chow diet and housed them in microisolator cages under specific pathogen-free conditions at the Center for Comparative Medicine, UConn Health. We used 6- to 24-week-old male and female mice in our experiments.

### Mouse monocyte isolation

We harvested bone marrow cells from mouse femurs and tibias and obtained monocytes by using the EasySep mouse monocyte isolation kit. Specifically, we added 50 μL·mL^-1^ each of rat serum and enrichment cocktails A and B to 1 × 10^8^ cells·mL^-1^ bone marrow cells and incubated at room temperature (RT) on a tube rotator for 5 min. We next added 75 μL·mL^-1^ vortexed magnetic particles to the cell suspension and incubated at RT for 3 min. Then, we used PBS to adjust the suspension volume to 2.5 mL and placed the tube into the EasySep Magnet for 3 min. After transferring the supernatant containing enriched monocytes to a new tube, we placed the new tube into the magnetic stand for an additional 2 min. After transferring the supernatant to another new tube and centrifuging at 500 × g for 5 min at RT, we resuspended the purified monocytes in RPMI-1640 medium without phenol red supplemented with 2% HSA, and used them within 4 h of isolation.

### Human peripheral blood monocyte isolation

Within 30 min of obtaining human blood samples, we enriched peripheral blood mononuclear cells (PBMCs) using density-gradient centrifugation with Ficoll-Paque Plus. Specifically, after diluting the blood to twice the volume with PBS, we layered the diluted blood onto Ficoll-Paque Plus at a 2:1 volume ratio and centrifuged at 400 × g at 20 °C for 35 min, resulting in PBMC enrichment at the plasma-Ficoll interface. After collecting PBMCs, we washed them twice with PBS by centrifugation at 300 × g for 10 min, then resuspended them in PBS.

Then, we used the EasySep human monocyte enrichment kit without CD16 depletion to purify monocytes. Specifically, we added 50 μL·mL^-1^ each of rat serum and enrichment cocktails to 5 × 10^7^ cells·mL^-1^ PBMCs and incubated at room temperature (RT) on a tube rotator for 10 min. We next added 50 μL·mL^-1^ vortexed magnetic particles to the cell suspension and incubated at RT for 5 min. Then, we used PBS to adjust the suspension volume to 2.5 mL and placed the tube into the EasySep Magnet for 3 min. After transferring the supernatant containing enriched monocytes to a new tube, we placed the new tube into the magnetic stand for an additional 2 min. After transferring the supernatant to another new tube and centrifuging at 500 × g for 5 min at RT, we resuspended the purified monocytes in RPMI-1640 medium without phenol red supplemented with 2% HSA, and used them within 4 h of isolation.

### Flow cytometry

We incubated 10^6^ cells·mL^-1^ purified human monocytes with 250 nM CytoD or DMSO vehicle control at RT for 10 min. To assess β_2_ integrin activation, we incubated cells with 2 μg·mL^-1^ each of AF647-conjugated mAb24 and DL550-conjugated KIM127 antibodies in the presence or absence of human CCL2 (100 ng·mL^-1^) at RT for 10 min. After antibody incubation, we fixed monocytes with 1% PFA at 4 °C for 10 min and washed twice with PBS before analysis. Fluorescence signals of cells were acquired using an LSRII flow cytometer (BD Biosciences, San Jose, CA) and analyzed with FlowJo software (version 10.6).

To assess β₂ integrin activation in mice, we collected 100 μL of peripheral blood retro-orbitally from human β₂ integrin knock-in mice. We incubated blood samples with 250 nM CytoD or DMSO vehicle control at RT for 10 min and stained them with FITC-conjugated mAb24, DL550-conjugated KIM127, and APC-conjugated anti-mouse CD115 antibody (2 μg·mL^-1^ each) in the presence or absence of 200 ng·mL^-1^ mouse CCL2 at RT for an additional 10 min. After antibody incubation, we fixed monocytes with 1% PFA at 4 °C for 10 min, lysed RBCs, and washed twice with PBS before analysis. Fluorescence signals of cells were measured on an LSRII cytometer and analyzed using FlowJo software. Monocytes were identified by gating on CD115-positive cells.

To evaluate monocyte viability, 10^6^ cells·mL^-1^ human monocytes treated with CytoD (250 nM) or vehicle control were stained with Annexin V-Pacific Blue (1:20) and SYTOX AADvanced (5 μM) for 30 min at RT. Following two PBS washes, samples were analyzed by flow cytometry. Untreated cells and cells exposed to 50% ethanol for 10 min at RT were included as negative and positive controls, respectively, for the gating of Annexin V- and SYTOX-negative viable cells.

For all integrin-related assays, antibody staining was performed before fixation, as fixation substantially reduces the accessibility of conformational epitopes due to integrin structural alterations.

### β_2_ integrin clustering assessed by STORM

STORM imaging solutions were prepared using pre-made buffer stocks. Buffer A contained 10 mM Tris (pH 8.0) and 50 mM NaCl, and Buffer B consisted of 50 mM Tris (pH 8.0), 10 mM NaCl, and 10% glucose. The glucose oxidase-catalase (GLOX) stock solution was prepared by dissolving 14 mg glucose oxidase and 1 mg catalase in 500 μL of Buffer A. A 1 M cysteamine (MEA) stock solution was prepared by dissolving 77 mg MEA in 1 mL of 0.25 N HCl.

Immediately before imaging, STORM imaging buffer was freshly assembled by mixing 2.3 μL of GLOX stock, 23.3 μL of 1 M MEA stock, and 206.6 μL of Buffer B. PBS in the imaging chamber containing the cell samples was then replaced with the freshly prepared STORM imaging buffer before image acquisition.

For STORM-based visualization and quantification of β₂ integrin clustering, μ-Slide 8-well glass-bottom chambers (Ibidi) were coated with 250 μL of 0.01% poly-L-lysine at 4°C overnight. The following day, wells were washed twice with ddH₂O, filled with 250 μL ddH₂O, and maintained at RT until use.

We pretreated 2 × 10^6^ cells·mL^-1^ purified human or mouse monocytes with CytoD (250 nM) or DMSO vehicle control at RT for 10 min. Human monocytes were stained with AF647-conjugated mAb24 and DL550-conjugated KIM127 antibodies (2 μg·mL^-1^ each) to label activated β₂ integrins in the presence of human CCL2 (100 ng·mL^-1^) at RT for 10 min. Mouse monocytes were incubated with AF647-conjugated anti-mouse CD18 antibody (2 μg·mL^-1^) together with mouse CCL2 (200 ng·mL^-1^) or PBS control at RT for 10 min. Following stimulation and labeling, cells were fixed with 1% paraformaldehyde and 0.05% glutaraldehyde at RT for 10 min.

After fixation, monocytes were washed twice with PBS, resuspended in PBS, and transferred into the pre-coated chamber wells following removal of ddH₂O. Chambers containing cells were centrifuged at 500 × g for 5 min at RT to immobilize cells on the bottom of the chamber. Immediately before imaging, PBS in the wells was replaced with freshly prepared STORM imaging buffer.

STORM images of the monocyte membrane surface contacting the chamber bottom were acquired using an Olympus iX83 inverted microscope equipped with an Abbelight SAFe Light module (405, 488, 532, and 640 nm lasers), Hamamatsu sCMOS fusion cameras, and a 100× NA 1.5 oil-immersion objective. Laser power at 532 nm or 640 nm was adjusted to 20% to yield approximately 20-50 fluorophore blinking events per 618 × 618-pixel frame (97 nm·pixel⁻¹; 60 × 60 μm² field of view) at appropriate threshold settings. Image acquisition was performed for 10,000 frames, yielding approximately 1,000-15,000 molecular localizations per cell.

Raw image sequences were processed using NEO Analysis software (Abbelight) with redundant cross-correlation-based drift correction 60. To minimize overcounting caused by repeated fluorophore blinking, consecutive localizations originating from the same fluorophore were merged by spatial-temporal tracking using the Octane analysis package (https://github.com/jiyuuchc/octane-core), with a maximum off-time threshold of five imaging frames.

β_2_ integrin clusters were identified using the Voronoi tessellation-based clustering algorithm^41^, ^43^ implemented in NEO Analysis. Parameters for cluster analysis included average density, density threshold, maximum inter-localization distance, and minimum number of localizations per cluster. As previously determined by the parameter scan 13, the results are not sensitive to changes in the average density and density threshold, which were therefore set to default values of 10 and 2, respectively. Our previous study 13 also determined a maximum distance of 25 nm and a minimum of 5 localizations per cluster as appropriate thresholds for our human monocyte analyses. For mouse monocytes, clustering was performed with an average density of 200, a density threshold of 2, a maximum distance of 25 nm, and a minimum of 5 localizations per cluster. Output metrics comprised the number of clusters, cluster density, and the number of β_2_ integrin localizations per cluster.

In parallel, we used the density-based spatial clustering of applications with noise (DBSCAN) algorithm, integrated into NEO Analysis, to quantify integrin clustering independently. DBSCAN parameters were set to an epsilon (Eps) value of 25 nm and a minimum point number (MinPts) of 5, consistent with the Voronoi tessellation settings, enabling the extraction of statistics, including the number of clusters, cluster density, and localizations per cluster.

### CORO1A membrane recruitment and actin cortex imaging

As described above, we prepared the glass-bottom μ-Slide 8-well chambers (Ibidi) by overnight coating with 0.01% poly-L-lysine at 4 °C. We pretreated 2 × 10^6^ cells·mL^-1^ purified human or mouse monocytes with CytoD (250 nM) or DMSO vehicle control at RT for 10 min, then stimulated them with 100 ng·mL^-1^ human CCL2 or 200 ng·mL^-1^ mouse CCL2, respectively, or PBS vehicle control at RT for 10 min. After fixation with 1% paraformaldehyde at 4 °C for 10 min, we washed cells twice with permeabilization buffer containing 5% goat serum.

To stain CORO1A, we incubated permeabilized monocytes with unconjugated rabbit anti-CORO1A antibody (1.25 μg·mL^-1^) diluted in permeabilization buffer with 5% goat serum at RT for 30 min. After washing the cells with PBS, we incubated them with an AF647-conjugated secondary antibody for 30 min at RT. Following fixation with 1% paraformaldehyde and 0.05% glutaraldehyde at RT for 10 min and two washes with PBS, we resuspended monocytes in PBS, transferred them to the poly-L-lysine-coated chamber wells, and immobilized them by centrifugation at 500 × g at RT for 5 min.

For F-actin visualization, we incubated permeabilized monocytes with AF568-conjugated phalloidin at RT for 30 min. Following fixation with 1% paraformaldehyde and 0.05% glutaraldehyde at RT for 10 min and two washes with PBS, we resuspended monocytes in PBS and immobilized them in the chamber wells as described above.

Epifluorescence images of transverse monocyte sections were acquired using an Olympus iX83 inverted microscope equipped with an Abbelight SAFe Light module (405, 488, 532, and 640 nm lasers), Hamamatsu sCMOS Fusion cameras, and a 100× NA 1.5 oil-immersion objective. In these images, the fluorescence intensities of CORO1A and F-actin were used as surrogates for their relative molecular abundances.

We assessed actin cortex formation and CORO1A membrane recruitment by using radial profile analysis. Specifically, radial intensity distributions were quantified with the Radial Profile plugin (https://imagej.net/ij/plugins/radial-profile.html) in Fiji-ImageJ, plotting fluorescence intensity distributions as a function of distance from the cell center, thereby enabling measurement of fluorescence redistribution from the cytoplasmic compartment toward the cell periphery following chemokine stimulation.

In parallel, we independently assessed actin cortex formation and CORO1A membrane recruitment by measuring the percentage of molecules expressed proximal to the cell membrane using Fiji-ImageJ (v2.0). Specifically, we used the circular selection tool to define regions corresponding to the whole cell and the cytoplasmic compartment, with the cytoplasmic region drawn as a concentric circle approximately 0.5 μm smaller in radius than the whole-cell region to account for the spatial resolution of epifluorescence imaging. We measured the accumulated fluorescence intensity and calculated the percentage of membrane-associated F-actin or CORO1A as [1 - (cytoplasmic fluorescence intensity / whole-cell fluorescence intensity)] × 100%.

### Co-immunoprecipitation and western blot

To examine the interaction between CORO1A and β_2_ integrins, we immunoprecipitated CD18-associated protein complexes from human monocytes and analyzed them by immunoblot. Magnetic beads used for the CD18 pull-down were prepared one day before the experiments. Specifically, 90 μL of streptavidin magnetic beads were incubated with 10 μL of 0.5 mg·mL^-1^ biotinylated anti-human CD18 monoclonal antibody at 4 °C overnight. Beads were subsequently washed twice with ice-cold 1× lysis buffer (50 mM Tris, 150 mM NaCl, 1 mM EDTA, 1% Triton X-100, supplemented with protease inhibitor cocktail at 1:500 dilution) using an EasySep magnet and stored on ice until use.

We incubated 4 × 10^6^ cells·mL^-1^ purified human monocytes with 250 nM CytoD or vehicle control at RT for 10 min, followed by stimulation with 100 ng·mL^-1^ human CCL2 for an additional 10 min. We lysed cells by adding an equal volume of 2× lysis buffer, vortexing, and incubating on ice for 30 min. Lysates were clarified by centrifugation at 12,000 rpm for 5 min at 4 °C to remove cellular debris. A fraction of each supernatant was retained as an input control before immunoprecipitation.

We incubated the supernatant with CD18 antibody-pre-coated magnetic beads at 4 °C overnight with gentle rotation. After incubation, we washed beads twice with ice-cold 1× lysis buffer using the EasySep magnet. Bound proteins were eluted, resolved by SDS-PAGE, and transferred onto nitrocellulose membranes.

The nitrocellulose membranes were blocked for approximately 30 min in tris-buffered saline containing 0.1% Tween-20 (TBST) and 5% non-fat milk, followed by incubation with primary antibodies diluted in TBST at 4 °C overnight. Rabbit anti-CORO1A antibody was used at a dilution of 1:500-1:1,000, and anti-CD18 monoclonal antibody was used at 1:1,000. After washing three times with TBST, membranes were incubated with horseradish peroxidase-conjugated secondary antibodies diluted 1:1,000 for CORO1A detection and 1:2,000 for CD18 detection for 1 h at RT. Protein bands were visualized using ECL Ultra substrate on an Azure 800 imaging system. Input lysates collected before immunoprecipitation were also analyzed by Western blot to assess CD18 and CORO1A expression levels.

### Co-localization assays

To quantify spatial associations among CD18, CORO1A, and F-actin, we labeled each pair on purified human or mouse monocytes and performed dual-color fluorescence imaging. We pretreated 2 × 10^6^ cells·mL^-1^ monocytes with 250 nM CytoD or vehicle control at RT for 10 min, then incubated them with AF647-conjugated anti-human or anti-mouse CD18 antibody, or left unstained where indicated, followed by stimulation with 100 ng·mL^-1^ human CCL2, 200 ng·mL^-1^ mouse CCL2, or PBS control at RT for 10 min. We subsequently fixed cells with 1% paraformaldehyde for 10 min and washed them twice with permeabilization buffer containing 5% goat serum.

For CD18/CORO1A or CORO1A/F-actin dual-color staining, permeabilized monocytes were incubated with unconjugated anti-CORO1A antibody (1.25 μg·mL^-1^) in permeabilization buffer containing 5% goat serum at RT for 30 min, whereas CD18/F-actin dual-color staining omitted CORO1A antibody incubation. For conditions requiring F-actin labeling, cells were incubated with AF568-conjugated phalloidin (0.44 μM) during this step. After one PBS wash, cells were incubated with AF568- or AF647-conjugated secondary antibodies to label primary anti-CORO1A antibody, as appropriate, at RT for 30 min, whereas CD18/F-actin dual-color staining omitted secondary antibody incubation. Samples were then fixed with 1% paraformaldehyde and 0.05% glutaraldehyde for 10 min. Following two PBS washes, cells were resuspended in PBS and immobilized onto poly-L-lysine-coated chamber wells as described above.

Dual-color epifluorescence images of transverse monocyte sections were first acquired using an Olympus iX83 inverted microscope equipped with an Abbelight SAFe Light module (405, 488, 532, and 640 nm lasers), Hamamatsu sCMOS Fusion cameras, and a 100× NA 1.5 oil-immersion objective. Subsequently, sequential dual-color STORM imaging was performed on the same samples after replacing PBS with freshly prepared STORM imaging buffer. STORM images were acquired from the monocyte membrane region contacting the chamber bottom, using identical microscope settings and acquisition parameters as described above. Briefly, laser power at 532 nm or 640 nm was adjusted to approximately 20% to yield 20-50 blinking events per 618 × 618-pixel frame, and image acquisition was performed for 10,000 frames, yielding ~1,000-25,000 molecular localizations per cell.

STORM image reconstruction was carried out using NEO Analysis software with redundant cross-correlation-based drift correction 60. For sequential dual-color STORM datasets, the drift-correction table generated from the first channel was imported during processing of the second channel to ensure spatial alignment between channels. Molecular co-localization was quantified using coordinate-based co-localization (CBC) analysis 61 implemented in NEO Analysis, reporting the fraction of CD18 molecules co-localized with CORO1A and vice versa. As previously established, CBC results were insensitive to molecular localization density 13; this was verified by randomly removing 50% of localization events from both channels and comparing CBC outputs before and after the reduction.

Co-localization in epifluorescence images was independently quantified using the JACoP plugin 62 in Fiji-ImageJ (v2.0), applying Pearson's correlation coefficient 63 to assess locational correlation between fluorescence channels.

### Statistical analysis

We used GraphPad Prism (version 9.4.1; GraphPad Software) for all statistical analyses. Specifically, we performed the Mann-Whitney U test to compare differences among experimental groups in most experiments, except for co-immunoprecipitation results, for which we performed paired Student's t-tests. Statistical significance was identified when p-values were <0.05.

## Supplementary Material

Supplementary figures.

## Figures and Tables

**Figure 1:**
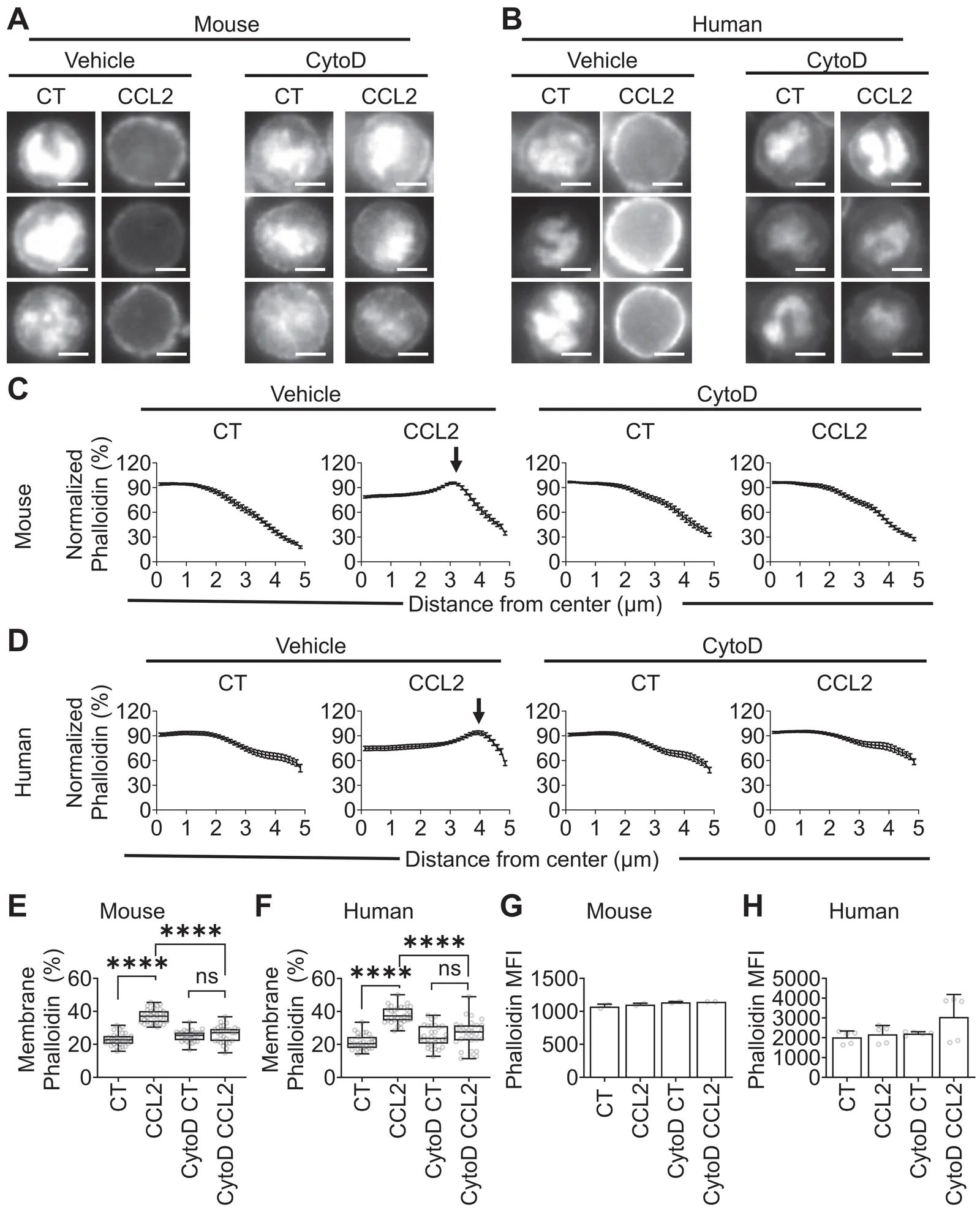
** CytoD inhibits monocyte actin cortex formation but not total actin polymerization. (A-B)** Representative epifluorescence transverse images of mouse (**A**) or human (**B**) monocytes stimulated by CCL2 (200 ng·mL^-1^ for mouse and 100 ng·mL^-1^ for human) or vehicle control showing F-actin (phalloidin) localization. Monocytes were pre-incubated with 250 nM Cytochalasin D (CytoD) or vehicle control. Scale bars are 5 μm. **(C-D)** Radial profile analysis of phalloidin fluorescence intensity in epifluorescence transverse images of mouse (**C**) or human (**D**) monocytes. Mean±SEM, n = 20 cells from 3 individual experiments. **(E-F)** Quantifications of the mouse (**E**) or human (**F**) monocyte epifluorescence transverse images showing the percentage of F-actin localized to the cell membrane. Boxplots (median, 25-75% range boxes, 0-100% range bars), n = 30 cells from 3 individual experiments. ns, not significant (*p* > 0.05), ****p < 0.0001 by Mann-Whitney unpaired non-parametric test. **(G-H)** Phalloidin median fluorescence intensity (MFI) of mouse (**G**) or human (**H**) monocytes assessed by flow cytometry, showing total actin polymerization. Mean±SD, n = 2 and 5 individual experiments in **G** and **H**, respectively.

**Figure 2:**
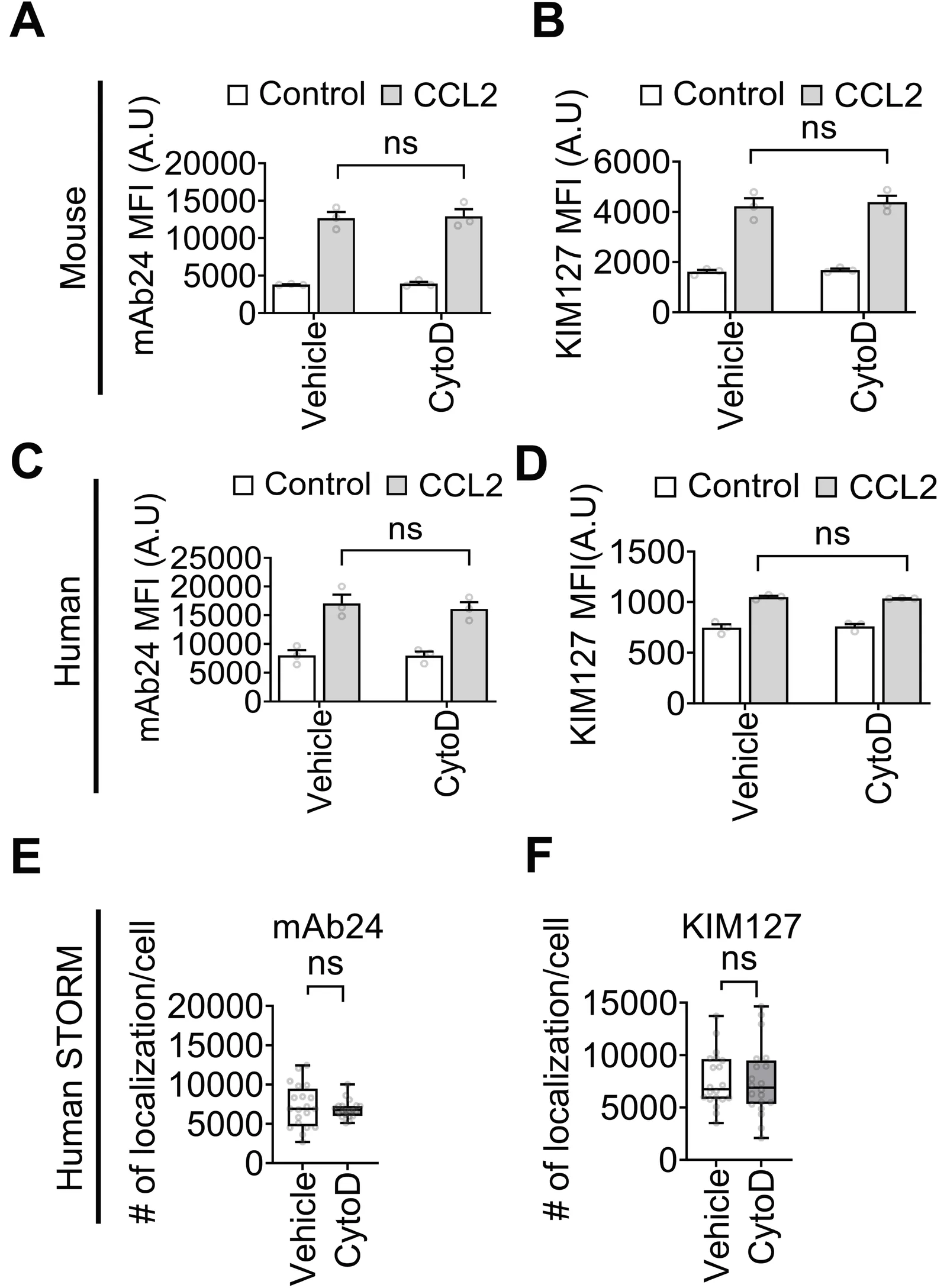
** Inhibition of actin cortex formation does not affect monocyte β_2_ integrin activation. (A-B)** Peripheral blood samples of human β_2_ integrin knock-in mice were incubated with 250 nM CytoD or vehicle control. β_2_ integrin activation on monocytes (CD115+) was quantified by flow cytometry using conformation-specific antibodies mAb24 (**A**) and KIM127 (**B**) after stimulated with mouse CCL2 (200 ng·mL^-1^) or vehicle control. Means ± SEM, n = 3 individual experiments. **(C-D)** Purified human blood monocytes were incubated with 250 nM CytoD or vehicle control. β_2_ integrin activation on monocytes was quantified by flow cytometry using conformation-specific antibodies mAb24 (**C**) and KIM127 (**D**) after being stimulated with human CCL2 (100 ng·mL^-1^) or vehicle control. Means ± SEM, n = 3 individual experiments. **(E-F)** Purified human monocytes were incubated with 250 nM CytoD or vehicle control. The number of mAb24 (**E**) and KIM127 (**F**) localizations per cell after CCL2 (100 ng·mL^-1^) stimulation was quantified by STORM imaging. Boxplots (median, 25-75% range box, and 0-100% range bars), n = 20 cells from four individual experiments. MFI, median fluorescence intensity. ns, not significant (*p* > 0.05) by Mann-Whitney unpaired non-parametric test.

**Figure 3:**
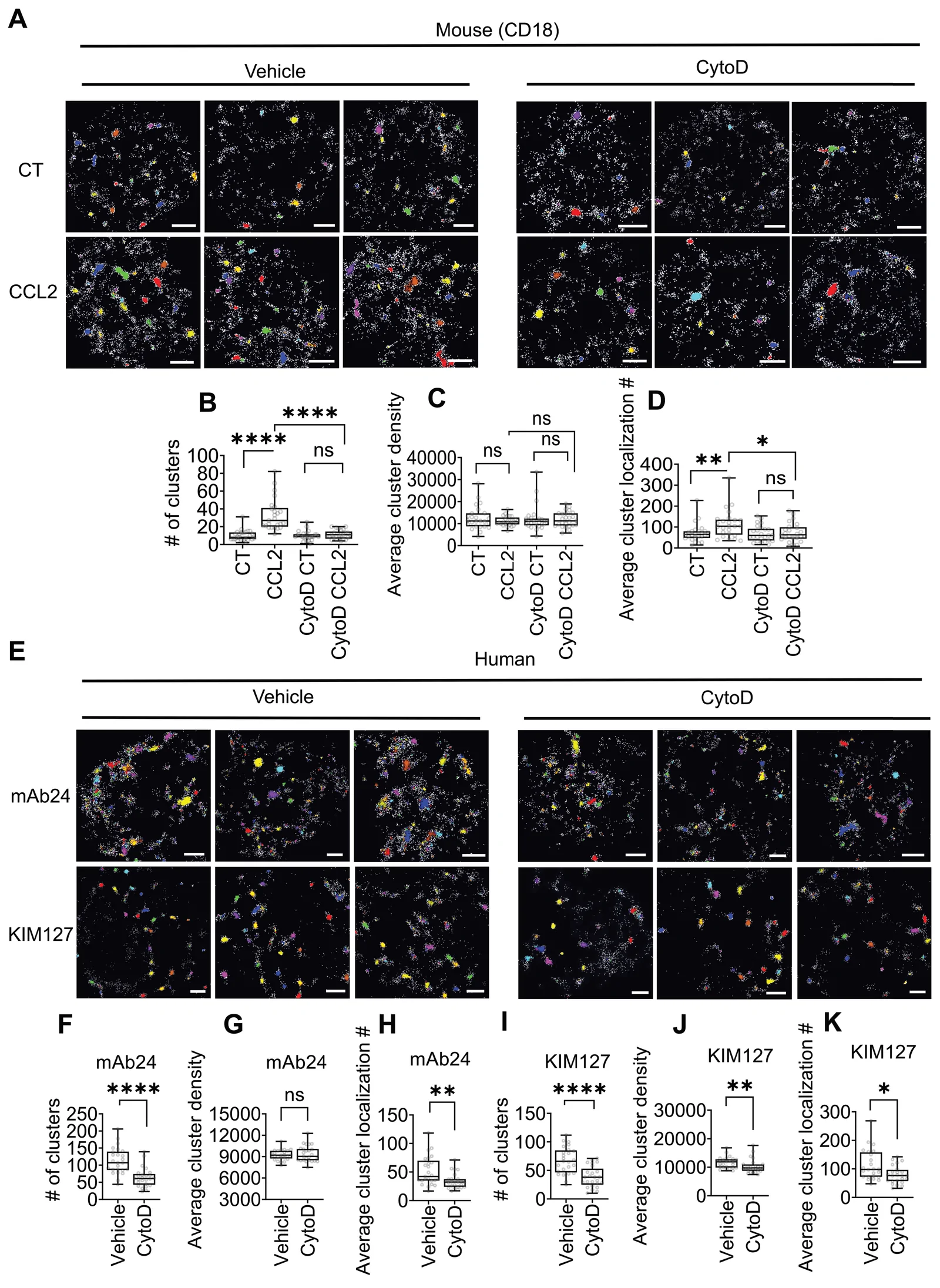
** Inhibition of actin cortex formation limits monocyte β_2_ integrin clustering. (A)** Representative STORM images of β_2_ integrin (CD18) clusters on the surface of mouse monocytes (the contact area with the coverslip). Mouse monocytes were incubated with 250 nM CytoD or vehicle control, then stimulated with CCL2 (200 ng/mL) or vehicle control (CT). **(B-D)** The number of clusters (**B**), the average cluster density (**C**), and the average cluster localization number of each cell (**D**) in STORM images. n = 25 cells from four individual experiments. **(E)** Representative STORM images of activated β_2_ integrin (mAb24 or KIM127) clusters on the surface of CCL2-stimulated human monocytes pre-incubated with 250 nM CytoD or vehicle control. **(F-K)** The number of activated β_2_ integrin clusters (**F**, **I**), average cluster density (**G**, **J**), and average cluster localization number of each cell (**H**, **K**) in STORM images. n = 25 cells from four individual experiments. Activated β_2_ integrins were labeled with conformation-specific antibodies mAb24 (**F-H**) or KIM127 (**I-K**). The clusters were labeled with different colors to distinguish adjacent clusters in** A** and** E**. Scale bars are 1 μm. Boxplots (median, 25-75% range boxes, 0-100% range bars) in **B-D** and **F-K**. *p < 0.05; **p < 0.01; ****p < 0.0001 by Mann-Whitney unpaired non-parametric test.

**Figure 4:**
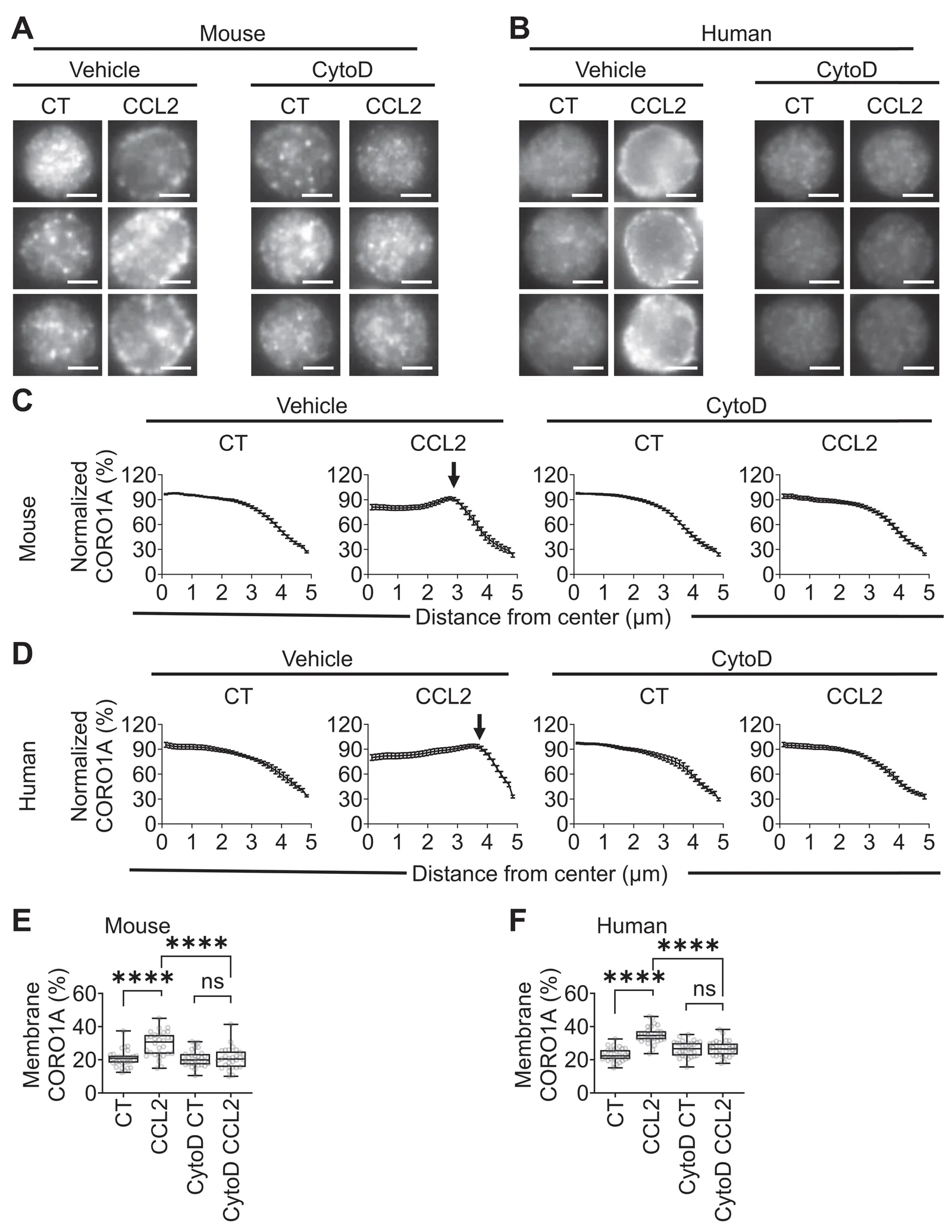
** Actin cortex-deficient monocytes cannot recruit CORO1A to the cell membrane. (A-B)** Representative epifluorescence transverse images of mouse (**A**) or human (**B**) monocytes stimulated by CCL2 (200 ng·mL^-1^ for mouse and 100 ng·mL^-1^ for human) or vehicle control showing CORO1A localization. Monocytes were pre-incubated with 250 nM CytoD or vehicle control. Scale bars are 5 μm. **(C-D)** Radial profile analysis of CORO1A fluorescence intensity in epifluorescence transverse images of mouse (**C**) or human (**D**) monocytes. Mean±SEM, n = 20 cells from 3 individual experiments. **(E-F)** Quantifications of the mouse (**E**) or human (**F**) monocyte epifluorescence transverse images showing the percentage of CORO1A localized to the cell membrane. Boxplots (median, 25-75% range boxes, 0-100% range bars), n = 30 cells from 3 individual experiments. ns, not significant (*p* > 0.05), ****p < 0.0001 by Mann-Whitney unpaired non-parametric test.

**Figure 5:**
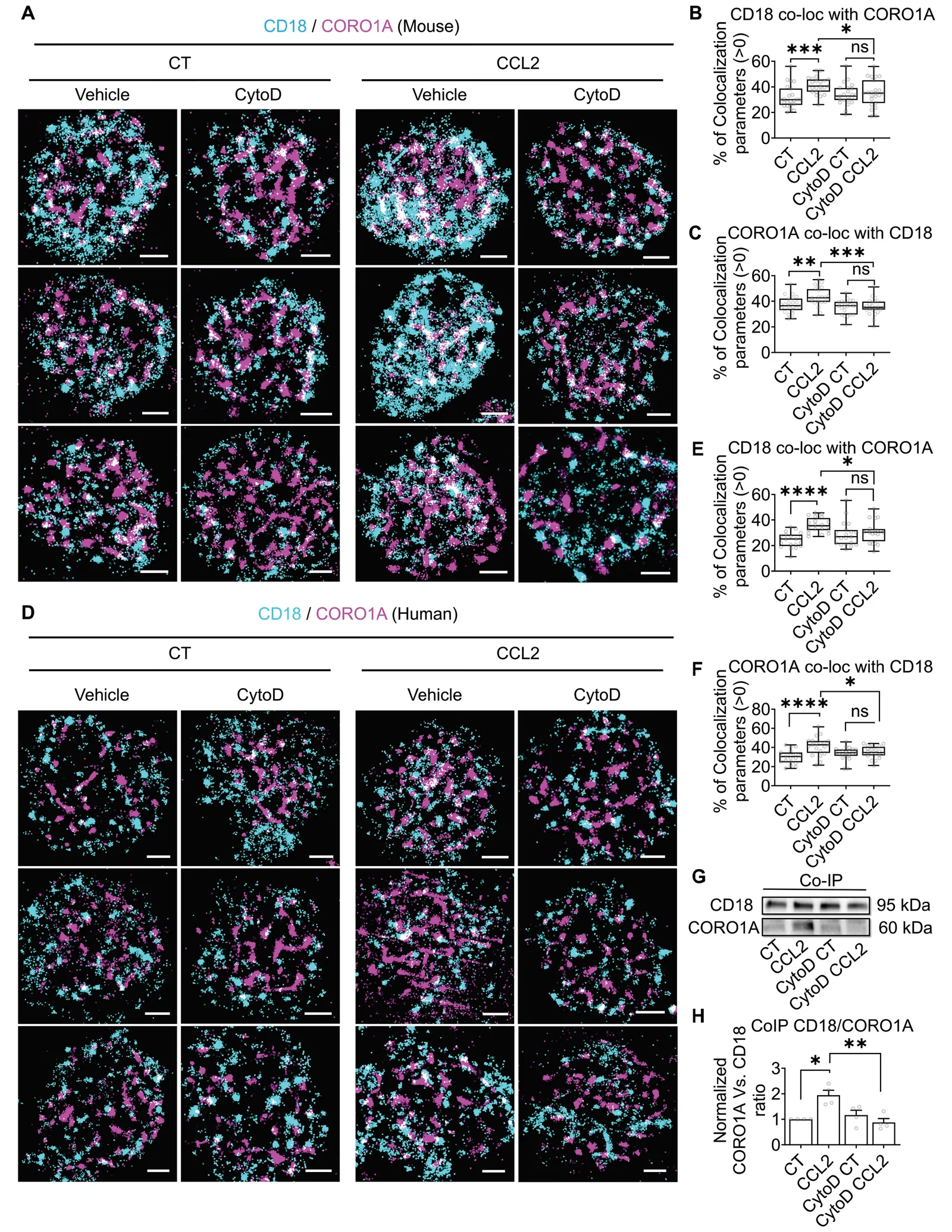
** Actin cortex-deficient monocytes exhibit defects in recruiting CORO1A to β_2_ integrins. (A)** Representative STORM images of β_2_ integrin (CD18, cyan) and CORO1A (magenta) on the surface of mouse monocytes (the contact area with the coverslip). Mouse monocytes were incubated with 250 nM CytoD or vehicle control, followed by stimulation with mouse CCL2 (200 ng·mL^-1^) or vehicle control (CT). Scale bars are 1 μm. **(B-C)** Coordinate-based co-localization analysis of STORM images showing the percentage of CD18 that co-localizes with CORO1A (**B**) and the percentage of CORO1A that co-localizes with CD18 (**C**). **(D)** Representative STORM images of β_2_ integrin (CD18, cyan) and CORO1A (magenta) on the surface of human monocytes. Human monocytes were pre-incubated with 250 nM CytoD or vehicle control and stimulated with human CCL2 (100 ng·mL^-1^) or vehicle control (CT). Scale bars are 1 μm. **(E-F)** Coordinate-based co-localization analysis of STORM images showing the percentage of CD18 that co-localizes with CORO1A (**E**) and the percentage of CORO1A that co-localizes with CD18 (**F**). Boxplots (median, 25-75% range boxes, 0-100% range bars). n ≥ 19 cells from four (**B** and** C**) and three (**E** and** F**) individual experiments. ns, not significant (*p* > 0.05), **p < 0.01; ***p < 0.001; ****p < 0.0001, by Mann-Whitney unpaired non-parametric test. **(G-H)** Human monocytes were pre-incubated with 250 nM CytoD or vehicle control and then stimulated with CCL2 (100 ng·mL^-1^) or vehicle control (CT). Cell lysates were used to immunoprecipitate the CD18-binding component. Representative western blot images (**G**) and quantifications (**H**) of CD18 and CORO1A amounts in the immunoprecipitated component. The CORO1A amount in **H** was normalized by the CD18 amount. Mean±SEM, n = 4 individual experiments (8 donors, pooled samples from two donors in each experiment). *p < 0.05; **p < 0.01 by paired Student's t-test.

**Figure 6:**
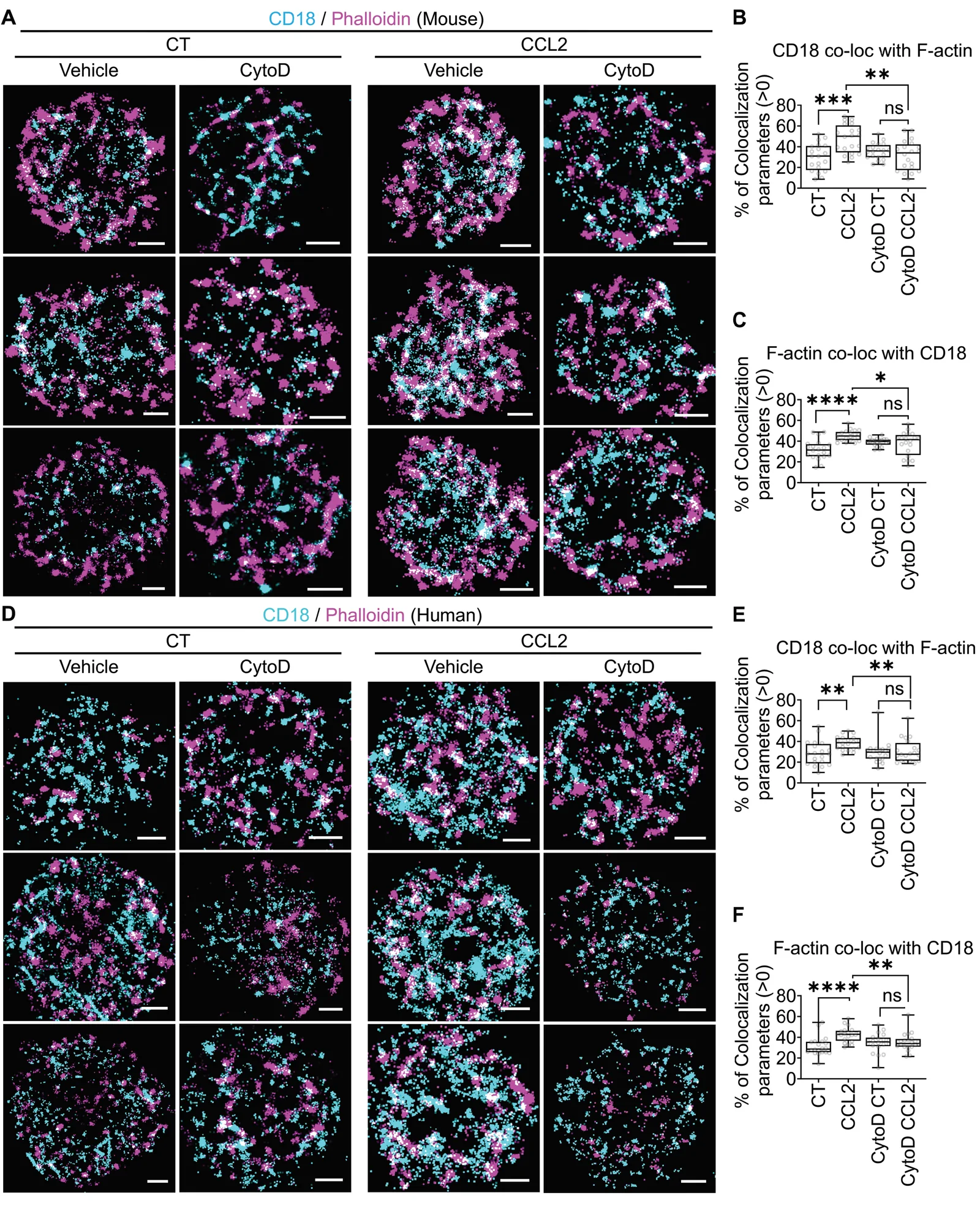
** Actin cortex-deficient monocytes have a defect in actin/β_2_ integrin co-localization. (A)** Representative STORM images of β_2_ integrin (CD18, cyan) and F-actin (phalloidin, magenta) on the surface of mouse monocytes (the contact area with the coverslip). Mouse monocytes were incubated with 250 nM CytoD or vehicle control and stimulated with mouse CCL2 (200 ng·mL^-1^) or vehicle control (CT). Scale bars are 1 μm. **(B-C)** Coordinate-based co-localization analysis of STORM images showing the percentage of CD18 that co-localizes with F-actin (**B**) and the percentage of F-actin that co-localizes with CD18 (**C**). **(D)** Representative STORM images of β_2_ integrin (CD18, cyan) and F-actin (phalloidin, magenta) on the surface of human monocytes. Human monocytes were pre-incubated with 250 nM CytoD or vehicle control and stimulated with human CCL2 (100 ng·mL^-1^) or vehicle control (CT). Scale bars are 1 μm. **(E-F)** Coordinate-based co-localization analysis of STORM images showing the percentage of CD18 that co-localizes with F-actin (**E**) and the percentage of F-actin that co-localizes with CD18 (**F**). Boxplots (median, 25-75% range boxes, 0-100% range bars). n = 20 cells from three (**B** and** C**) and four (**E** and** F**) individual experiments. ns, not significant (*p* > 0.05), *p < 0.05; **p < 0.01; ***p < 0.001; ****p < 0.0001 by Mann-Whitney unpaired non-parametric test.

## Data Availability

The datasets used and/or analyzed during the current study are available from the corresponding author on reasonable request.

## Abbreviations

AF: Alexa Fluor
APC: Allophycocyanin
CBC: Coordinate-based co-localization
CCL2: C-C motif chemokine ligand 2
CD18: Integrin β2 subunit
CFTR: Cystic fibrosis transmembrane conductance regulator
CytoD: Cytochalasin D
DBSCAN: Density-based spatial clustering of applications with noise
FBS: Fetal bovine serum
GPCR: G protein-coupled receptor
HSA: Human serum albumin
ICAM-1: Intercellular adhesion molecule 1
IRB: Institutional Review Board
IACUC: Institutional Animal Care and Use Committee
MEA: Cysteamine
PBS: Phosphate-buffered saline
PFA: Paraformaldehyde
STORM: Stochastic optical reconstruction microscopy
