# Supplementary material for: Actin cortex formation is required for integrin clustering but not activation during leukocyte adhesion
Source: Int J Biol Sci. 2026 Sep 10;22(15):8158–75. doi: 10.7150/ijbs.137102 (PMC13685610; doi:10.7150/ijbs.137102)
Supplement: Supplementary file 1 [file ijbsv22p8158s1.pdf]

Figure S1

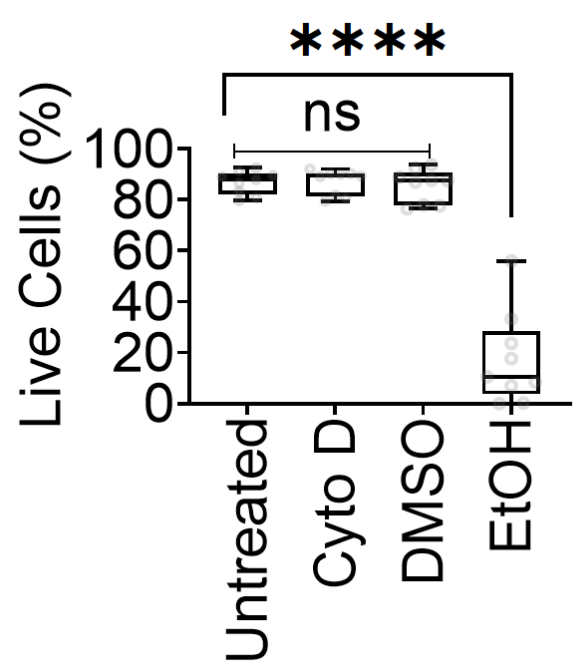

Fig. S1. Monocyte viability after Cytochalasin D (CytoD) treatment. Purified human monocytes were incubated with 250 nM CytoD or the DMSO vehicle control for 10 min, and the cell viability was assessed by quantifying Annexin V and SYTOX AADv87panced staining in flow cytometry. Untreated and 50% ethanol-treated (EtOH, RT, 10 min) monocytes were used as negative and positive controls, respectively, for the gating of Annexin V-SYTOX double-negative live cells. Boxplots (median, 25-75% range box, and 0-100% range bars), n = 9 repeats from 3 individual experiments. ns, not significant ($p > 0.05$), ****$p < 0.0001$ by the One Way ANOVA.

Figure S2

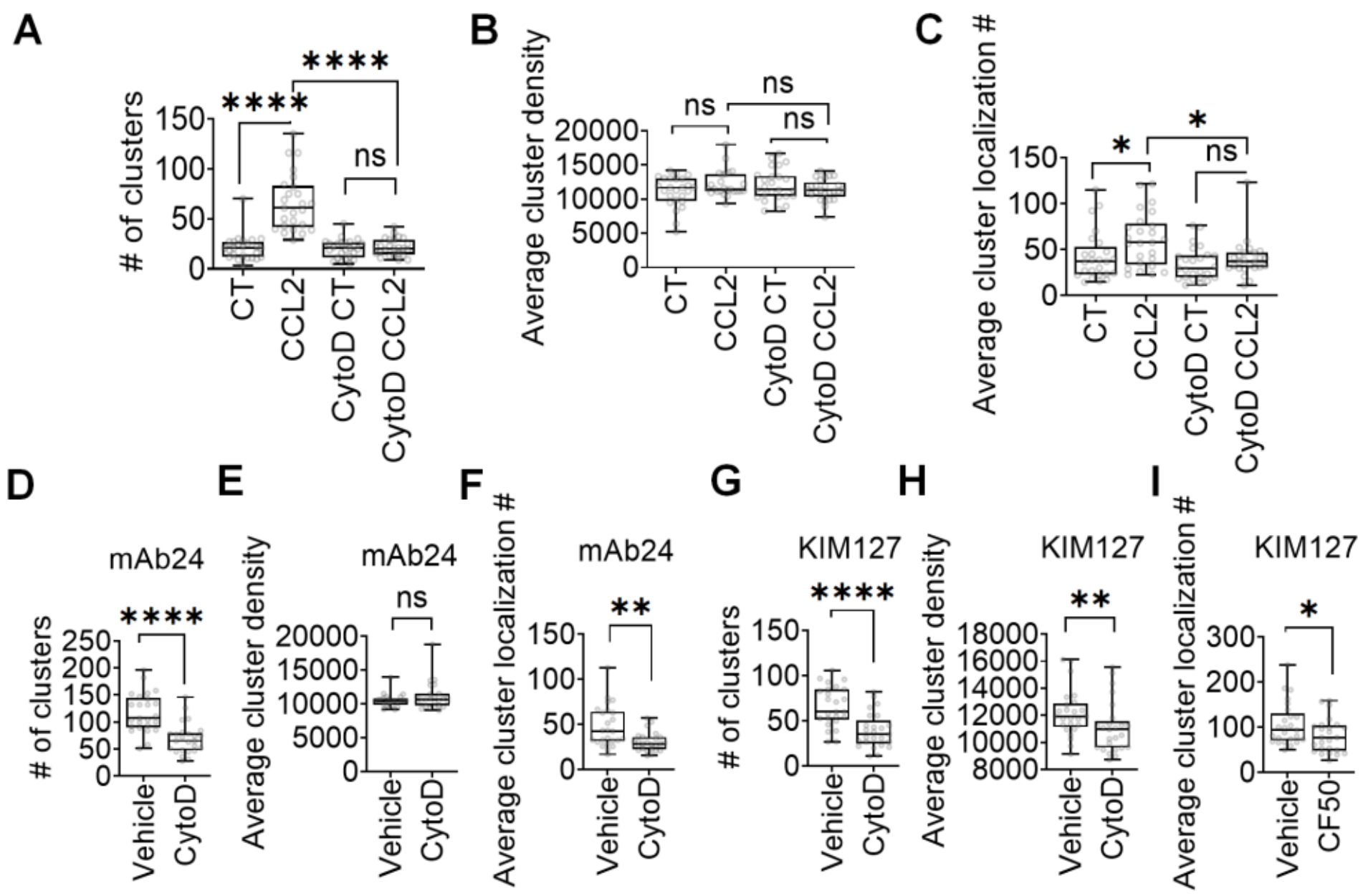

Fig. S2. β_2 integrin clustering analysis using DBSCAN. (A-C) Mouse monocytes were incubated with 250 nM CytoD or the vehicle control, followed by stimulation with mouse CCL2 (200 ng·mL^{-1}) or the vehicle control. STORM images of β_2 integrin clusters on the monocyte surface (the contact area with the coverslip) were analyzed by the DBSCAN algorithm. The number of clusters (**A**), the average cluster density (**B**), and the average cluster localization number of each cell (**C**) are shown. **(D-I)** Activated β_2 integrins of CCL2-stimulated human monocytes pre-incubated with 250 nM CytoD or the vehicle control were labeled with mAb24 (**D-F**) or KIM127 (**G-I**) antibodies. Clusters on the surface (the contact area with the coverslip) were imaged by STORM and were analyzed by the DBSCAN algorithm. The number of clusters (**D**, **G**), the average cluster density (**E**, **H**), and the average cluster localization number of each cell (**F**, **I**) were shown. Boxplots (median, 25-75% range boxes, 0-100% range bars). n = 25 cells from four individual experiments. *p < 0.05; **p < 0.01; ****p < 0.0001 by the Mann-Whitney U test.

Figure S3

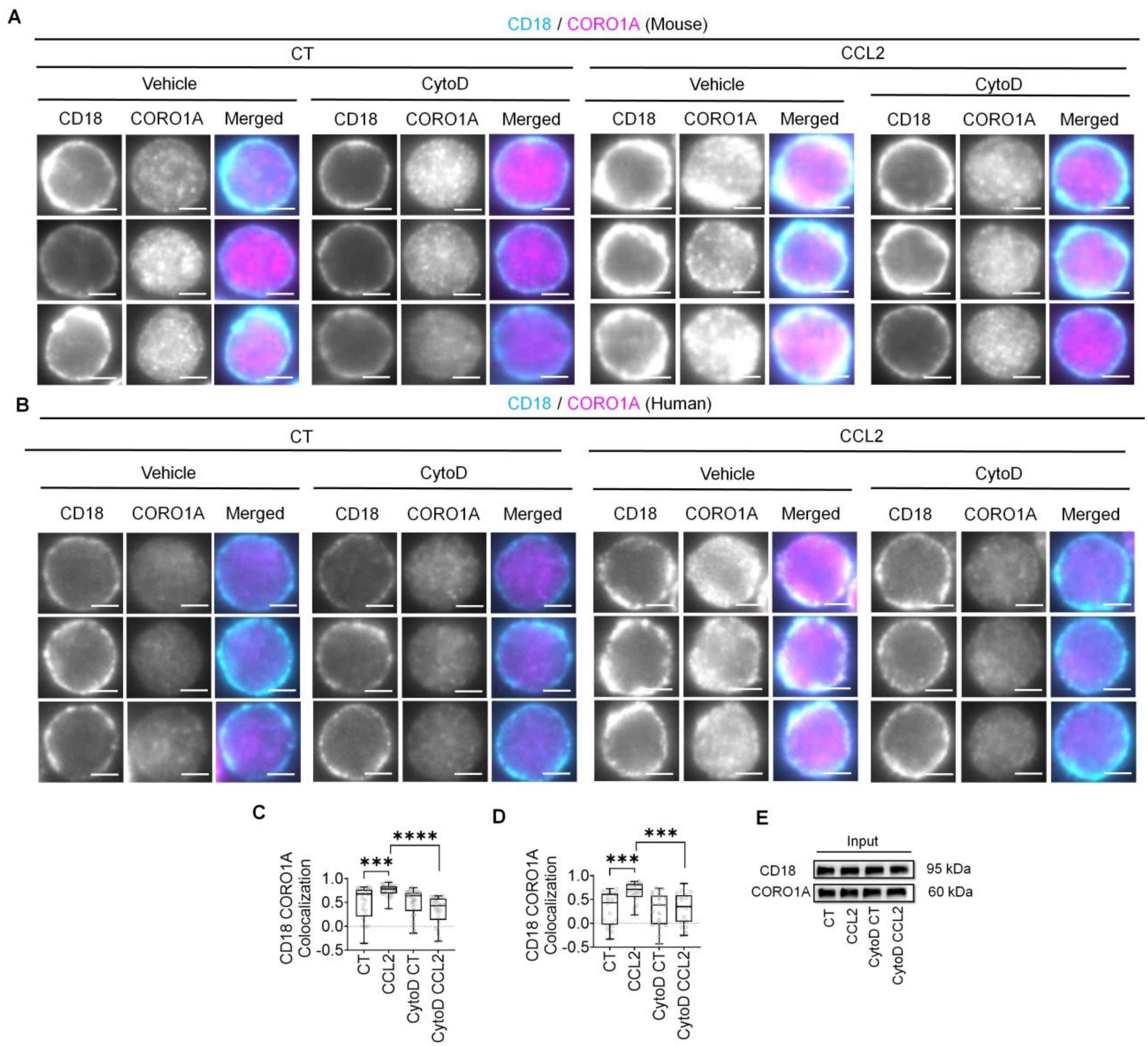

Fig. S3. Actin cortex-deficient monocytes exhibit defects in recruiting CORO1A to β$_2$ integrins. (A) Representative epifluorescence transverse images of β$_2$ integrin (CD18, cyan) and CORO1A (magenta) in mouse monocytes. Mouse monocytes were incubated with 250 nM CytoD or the vehicle control, followed by stimulation with mouse CCL2 (200 ng·mL^{-1}) or the vehicle control (CT). Scale bars are 5 μm. **(B)** Representative epifluorescence transverse images of β$_2$ integrin (CD18, cyan) and CORO1A (magenta) in human monocytes. Human monocytes were pre-incubated with 250 nM CytoD or the vehicle control and

stimulated with human CCL2 (100 ng·mL^{-1}) or the vehicle control (CT). Scale bars are 5 μm. **(C-D)** Pearson's correlation coefficient of CD18 and CORO1A in mouse (**C)** and human blood monocytes (**D**). Boxplots (median, 25-75% range boxes, 0-100% range bars). n = 25 (**C**) or 20 (**D**) cells from three (**D**) or four **(C**) individual experiments. ***p < 0.001; ****p < 0.0001 by the Mann-Whitney U test. **(E)** Representative western blot images show expression of CD18 and CORO1A in cell lysates before immunoprecipitation.

Figure S4

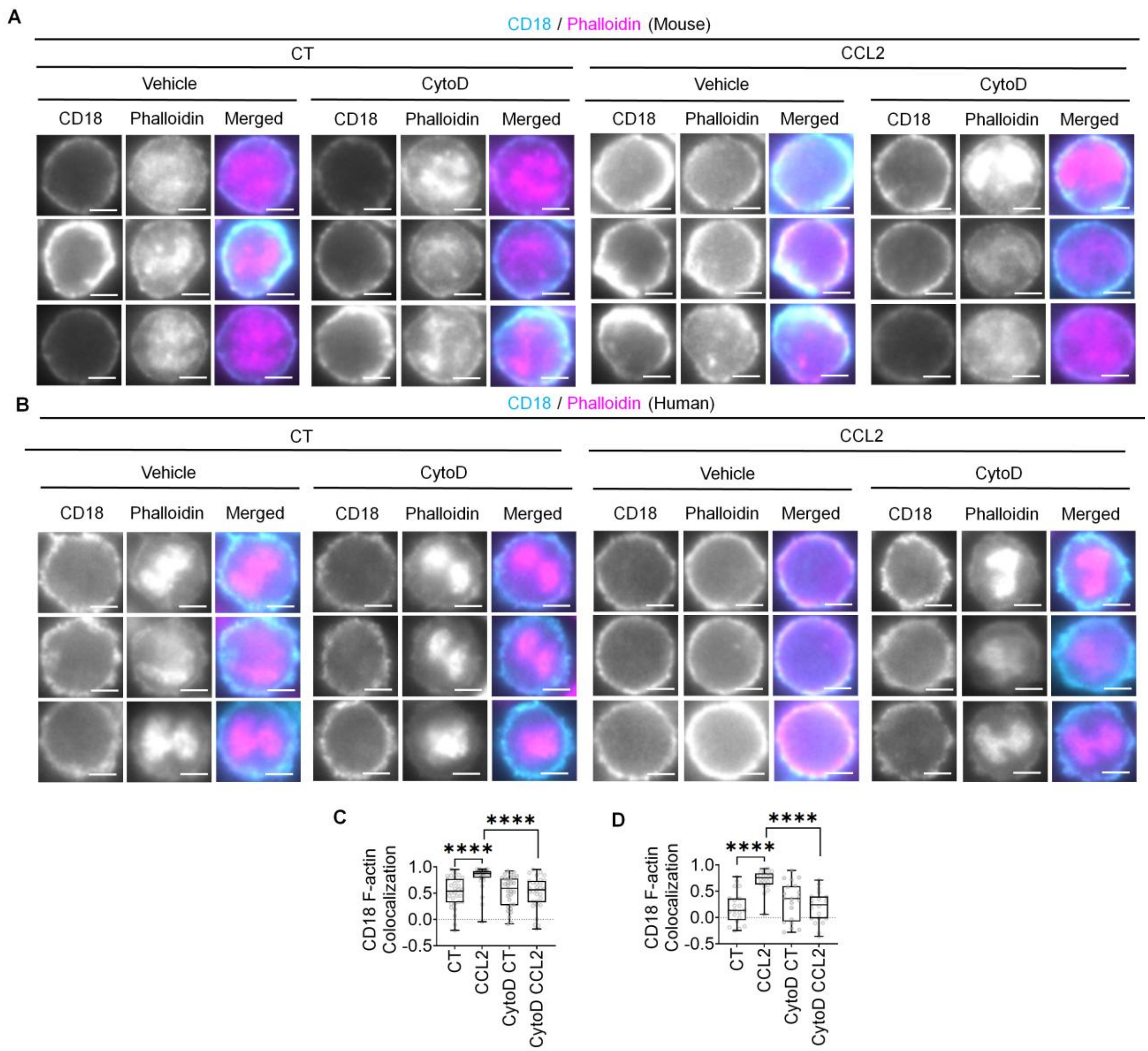

Fig. S4. Actin cortex-deficient monocytes have a defect in actin/β$_2$ integrin co-localization. (A) Representative epifluorescence transverse images of β$_2$ integrin (CD18, cyan) and F-actin (phalloidin, magenta) in mouse monocytes. Mice monocytes were incubated with 250 nM CytoD or the vehicle control and stimulated with mouse CCL2 (200 ng·mL^{-1}) or the vehicle control (CT). Scale bars are 5 µm. **(B)** Representative epifluorescence transverse images of β$_2$ integrin (CD18, cyan) and F-actin (phalloidin, magenta) in human monocytes. Human monocytes were pre-incubated with 250 nM CytoD or the vehicle

control and stimulated with human CCL2 (100 ng·mL^{-1}) or the vehicle control (CT). Scale bars are 5 μm. **(C-D)** Pearson's correlation coefficient of CD18 and F-actin (phalloidin) in mouse (**C**) and human blood monocytes (**D**). Boxplots (median, 25-75% range boxes, 0-100% range bars). n = 30 (**C**) or 20 (**D**) cells from three (**D**) or four (**C**) individual experiments. ****$p < 0.0001$ by the Mann-Whitney U test.

Figure S5

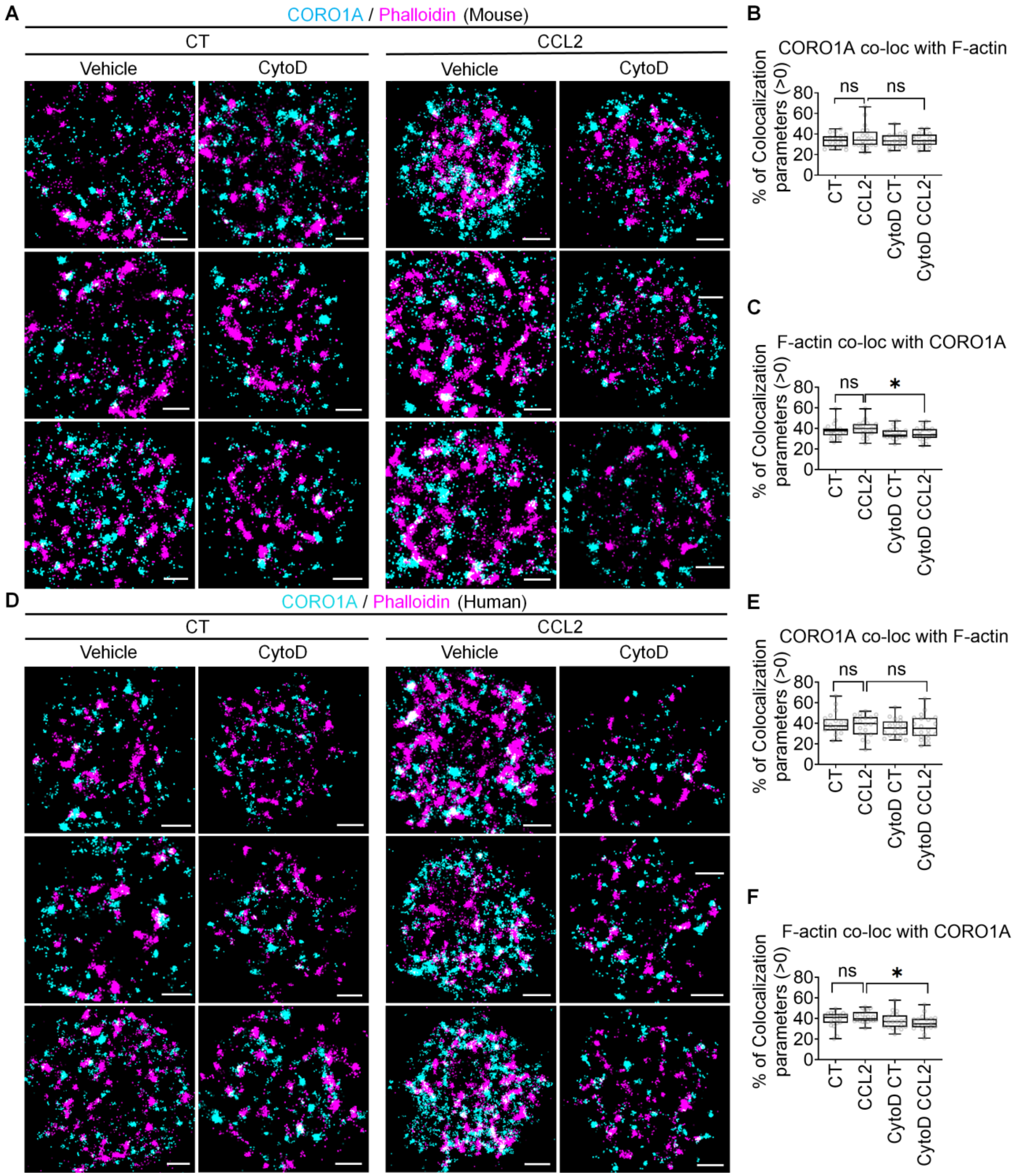

Fig. S5. Unaffected colocalization of CORO1A and F-actin in monocytes. (A) Representative STORM images of CORO1A (cyan) and F-actin (phalloidin, magenta) on the surface of mouse monocytes (the

contact area with the coverslip). Mouse monocytes were incubated with 250 nM CytoD or the vehicle control and stimulation with mouse CCL2 (200 $ng \cdot mL^{-1}$) or the vehicle control (CT). Scale bars are 1 μm. **(B-C)** Coordinate-based co-localization analysis of STORM images showing the percentage of CORO1A co-localizes with F-actin (**B**) and F-actin co-localizes with CORO1A (**C**). **(D)** Representative STORM images of CORO1A (cyan) and F-actin (phalloidin, magenta) on the surface of human monocytes. Human monocytes were pre-incubated with 250 nM CytoD or the vehicle control and stimulated with human CCL2 (100 $ng \cdot mL^{-1}$) or the vehicle control (CT). Scale bars are 1 μm. **(E-F)** Coordinate-based co-localization analysis of STORM images showing the percentage of CORO1A co-localizes with F-actin (**E**) and F-actin co-localizes with CORO1A (**F**). Boxplots (median, 25-75% range boxes, 0-100% range bars). n = 20 cells from three individual experiments. ns, not significant ($p > 0.05$), *$p < 0.05$; by the Mann-Whitney U test.

Figure S6

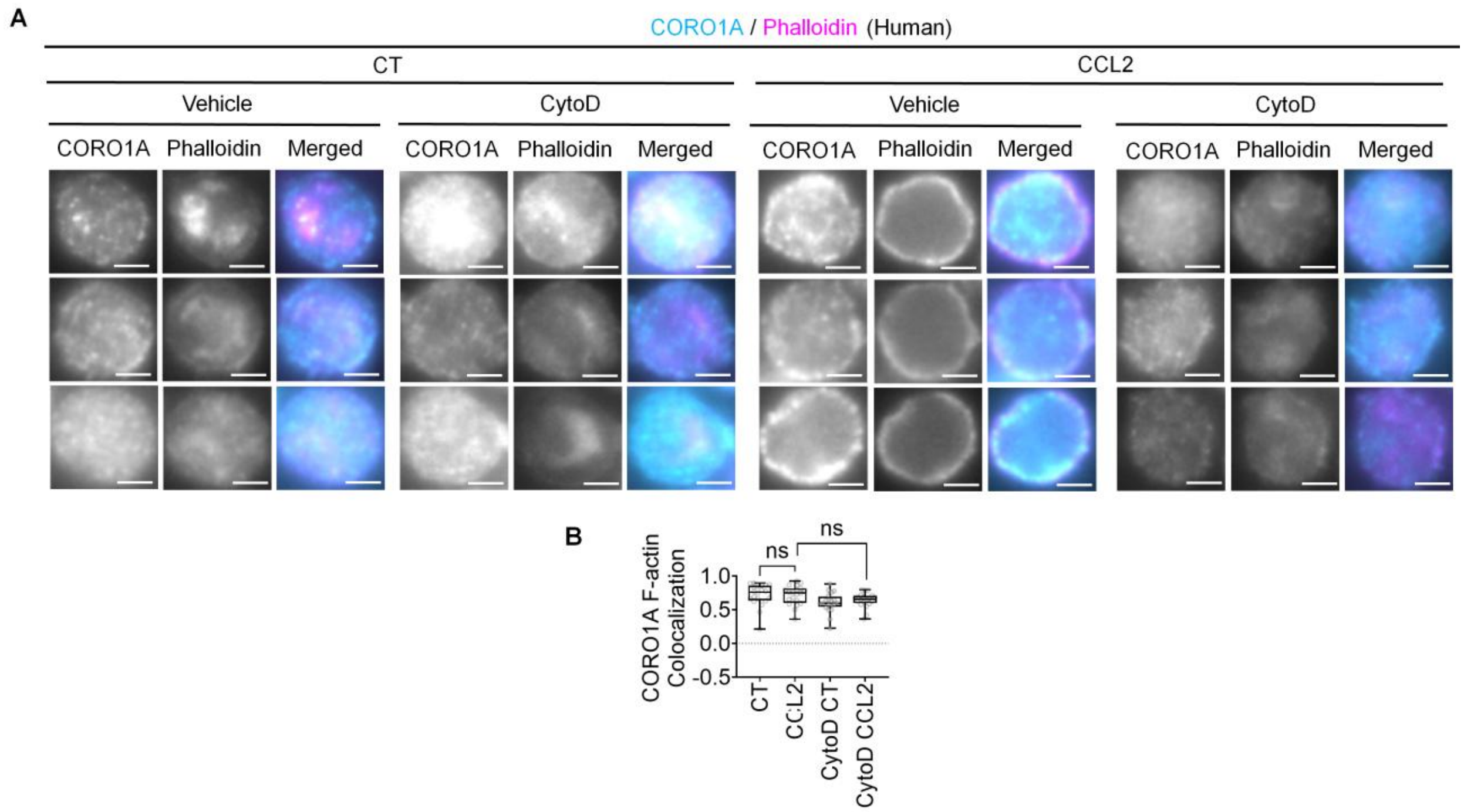

Fig. S6. Unaffected colocalization of CORO1A and F-actin in monocytes. (A) Representative epifluorescence transverse images of CORO1A (cyan) and F-actin (phalloidin, magenta) on human monocytes. Human monocytes were pre-incubated with 250 nM CytoD or the vehicle control and stimulated with human CCL2 (100 ng·mL^{-1}) or the vehicle control (CT). Scale bars are 5 µm. **(B)** Pearson's correlation coefficient of CORO1A and F-actin (phalloidin) in human monocytes. Boxplots (median, 25-75% range boxes, 0-100% range bars). n = 20 cells from three individual experiments. ns, not significant ($p > 0.05$) by the Mann-Whitney U test.

Figure S7

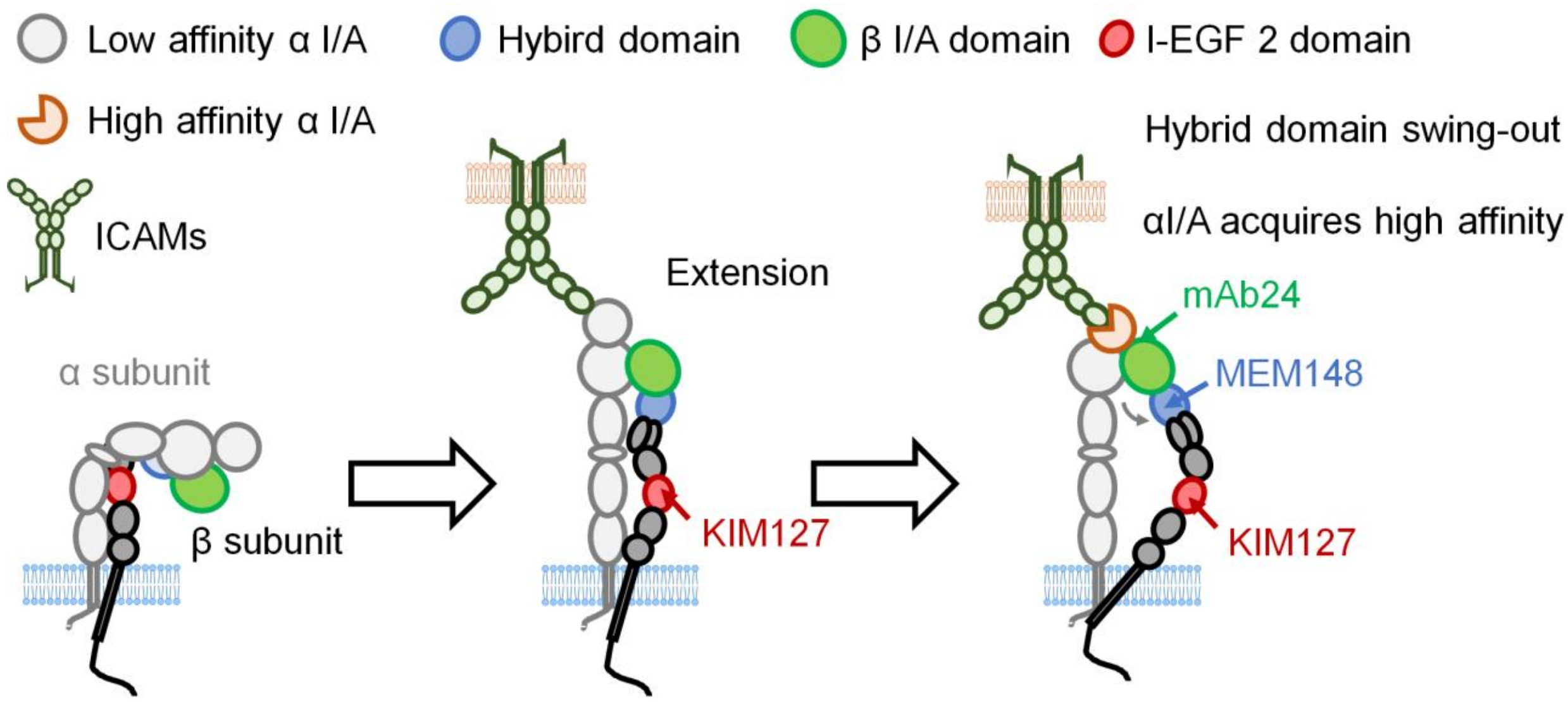

Fig. S7. Schematics showing the switch-blade model of integrin activation and binding sites of reporter antibodies on human β_2 integrins. β_2 integrins are heterodimers of α and β subunits. The resting state of β_2 integrins is bent and has low affinity for the ligand (e.g., ICAMs)-binding affinity. Upon inside-out activation signaling, the bent integrins will extend first, and the KIM127 binding epitope on the I-EGF 2 domain will be exposed. Then, the hybrid domain can swing out, which will expose the MEM148 binding epitope, and the β I/A domain will have conformational changes and acquire high affinity to an internal binding residue on the α I/A domain, causing the conformational changes of the α I/A domain to acquire high affinity to ligands. The mAb24 binding epitope will be exposed when the β I/A domain acquires high affinity.
